# Plant Metabolites Screening Identifies 7‐epi‐10‐Deacetyltaxol Inhibiting Cell Division by Interfering With the Cell Cycle and Microtubule Organization

**DOI:** 10.1002/advs.77835

**Published:** 2026-09-16

**Authors:** Yan‐Jie Zhang, Yu‐Xuan Bai, Geng G. Tian, Bin Zhang, Ding‐Yi Lu, Qing‐Ping Yao, Wen‐Hao Tian, Xiao‐Na Wei, Jing‐Ting Yang, Ze‐Yu Xiong, Yi Lu, Yu‐Tong Jiang, Chun‐Hui Cai, Yu‐Ming Lu, Xian‐Qing Jia, Li‐Li Jing, Bao‐Jun Yang, Ying‐Xin Qi, Xin‐Xin Han, Ji Wu, Wen‐Hui Lin

**Affiliations:** ^1^ State Key Laboratory of Microbial Metabolism Joint International Research Laboratory of Metabolic and Developmental Sciences School of Life Sciences and Biotechnology Shanghai Jiao Tong University Shanghai China; ^2^ Zhiyuan College Shanghai Jiao Tong University Shanghai China; ^3^ Key Laboratory for the Genetics of Development & Neuropsychiatric Disorders (Ministry of Education), Bio‐X Institutes Shanghai Jiao Tong University Shanghai China; ^4^ School of Agriculture and Biology Shanghai Jiao Tong University Shanghai China; ^5^ Key Laboratory of Fertility Preservation and Maintenance of Ministry of Education Ningxia Medical University Yinchuan China; ^6^ School of Pharmaceutical Sciences Shanghai Jiao Tong University Shanghai China; ^7^ Shanghai Lisheng Biotech Shanghai China; ^8^ Shanghai Collaborative Innovation Center of Agri‐Seeds/Joint Center for Single Cell Biology Shanghai Jiao Tong University Shanghai China; ^9^ Key Laboratory of Resource Biology and Biotechnology Western China Ministry of Education Provincial Key Laboratory of Biotechnology of Shaanxi Province College of Life Sciences Northwest University Xi'an China; ^10^ State Key Laboratory of Plant Cell and Chromosome Engineering Institute of Genetics and Developmental Biology Chinese Academy of Sciences Beijing China; ^11^ LiSheng Organ Regeneration X Lab East China Institute of Biotechnology Peking University Jiangsu China

**Keywords:** 7‐epi‐10‐deacetyltaxol, cell division, microtubule, organoid, plant screening system

## Abstract

Plants produce a considerable number of metabolites, and many of them appear to interfere with certain cellular processes such as cell division. For example, taxanes produced by *Taxus* are known as anti‐cancer drugs that stabilize microtubules and prevent the cell from dividing normally. Disorder of cell division also leads to abnormal growth and development in plants. It is worthy of consideration whether these metabolites have negative effects on plants per se. In this study, we developed an efficient screening system for identifying plant metabolites affecting rapeseed seedling root growth. One compound, 7‐epi‐10‐deacetyltaxol, inhibited tissue elongation by adversely altering cell division and elongation. And it was applied to plant cell lines, rat vascular smooth muscle cells, and human tumor organoids, resulting in similar cell division suppression. Transcriptome and proteomics analysis indicated the suppression in plant growth was through brassinosteroid‐regulated expression of cell cycle proteins, while cytological observation reveals microtubule morphology anomalies. Single‐nucleus transcriptome sequencing revealed inhibitory effects of ovarian cancer organoids. Together, our findings identified a universal cell division inhibitor and characterized its conserved function with respective regulatory mechanisms, providing new insights into what role “harmful” plant metabolites are playing in plants.

## Graphical abstract

In this study, an efficient screening system is developed for plant metabolites to find a compound, 7‐epi‐10‐deacetyltaxol, inhibits tissue elongation. It suppresses plant growth through brassinosteroid and microtubule pathways. It is later applied to animal cells and human tumor organoids, resulting in similar cell division suppression. These findings identified a universal cell division inhibitor and characterized its respective mechanisms.

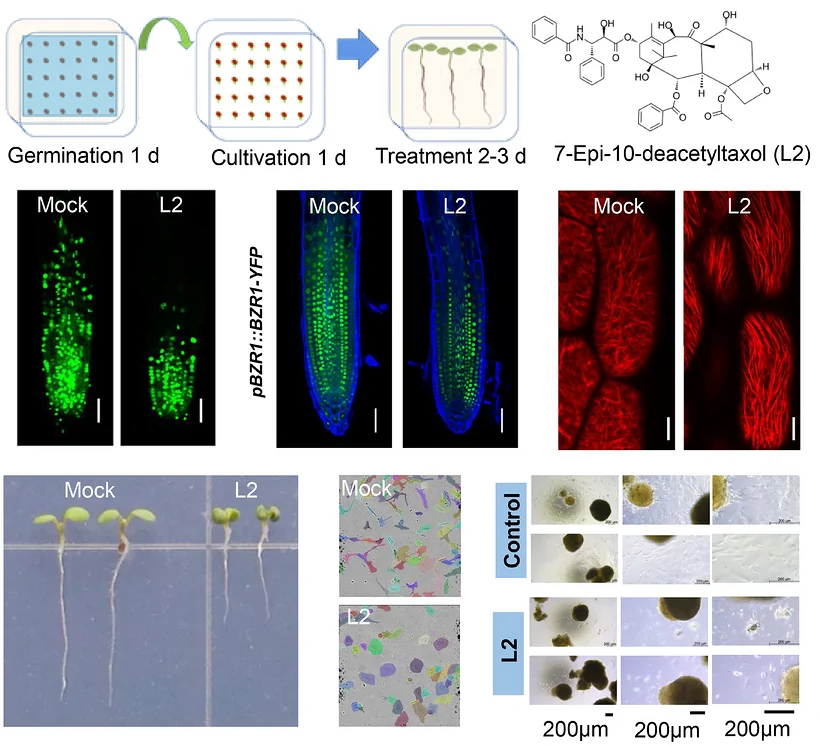

## 1 Introduction

Moderate and controlled cell division is fundamental to the growth and development of both plants and animals [1]. In plants, cell division not only determines cell number and organ size but also contributes to growth direction and morphogenesis, especially in organs exhibiting pronounced longitudinal growth, such as roots [2]. Studies have shown that plant cell division is regulated by an integrated cell cycle regulatory network centered on CDKs, cyclins, and E2F/RBR [1, 3], and hormonal signaling pathways such as auxin, cytokinin, and brassinosteroids [2, 4]. Simultaneously, the microtubule cytoskeleton and its role in organizing the spindle and phragmoplast are equally essential for chromosome segregation, cytokinesis, and the control of division orientation [5, 6].

Plants and animals share partially conserved components in the cell cycle, cytoskeleton, or other cellular events. This is supported by the fact that plenty of plant metabolites exhibit anti‐tumor activity. For example, paclitaxel, or taxanes, are a large group of anticancer drugs targeting microtubules. It was first isolated and characterized from the bark of the Pacific yew (*Taxus brevifolia*). Although over 40 000 works on their inhibitory mechanisms and synthesis have been published, explorations for novel mechanisms and synthesis of new taxanes derivatives with better performance and lower cytotoxicity are still being made. Notably, the overwhelming majority of taxane research conducted in plants focuses on improving taxane yield by cell culture. Although certain “by‐products” unveiled a corner of the in situ action mode of paclitaxel in plants, we still know little about the precise effect of paclitaxel and its derivatives on plant cells and why Taxus produces taxanes. These anti‐cancer plant metabolites remind us that we may apply plants for drug discovery based on those conserved mechanisms and components, with the requirement of strict verification.

Chemical genetics has become a key part of modern biological research, enabling the identification of small molecules that modulate organism growth and development. Chemical screening has been widely applied to search for molecules affecting animal growth at cellular and developmental levels for medical requirements. Basically, it involves treating certain cell lines or tissue of interest with a compound library and performing analysis of any chemical compounds that modulate biological processes to reveal therapeutic targets or develop novel drugs [7, 8]. In plants, chemical screening also provides aid for novel mechanisms relevant to multiple developmental processes, such as vacuolar sorting, flowering, pollen tube growth, circadian clock, root growth, and branching have been investigated on the basis of compounds found in chemical screening [7, 9, 10, 11, 12]. Moreover, the identification of compounds that function as receptor antagonists and thus modulate hormone functions has led to significant advances in phytohormone signaling research [9, 10, 13]. Some studies have also identified chemical tools that can manipulate plant stress resistance or disease resistance enhancement [14, 15, 16]. These researches suggest that chemical genetics or screening works as a powerful tool for plant developmental and molecular biology research.

Ovarian cancer remains one of the most lethal gynecological malignancies, posing a significant threat to women's health and survival. Despite considerable improvements in therapeutic strategies, the prognosis for patients with advanced‐stage ovarian cancer remains poor, with a 5‐year survival rate of only 47% [17]. This high mortality rate is due to multiple factors, including late‐stage diagnosis, tumor heterogeneity, and chemoresistance. While recent therapeutic approaches, including paclitaxel‐platinum combination chemotherapy, intraperitoneal drug delivery, and risk‐reducing surgery, are promising treatments for epithelial ovarian cancer, chemoresistance continues to limit overall survival rates [18]. Therefore, there is a need for novel drug alternatives for treating ovarian cancer.

In the biomedical field, organoids are invaluable materials for modeling diseases and discovering new therapeutics [19]. On September 29, 2022, the US Senate unanimously passed the FDA Modernization Act 2.0, which has the goal of eliminating federal mandates for animal testing of new and generic drugs to significantly decrease the need for animal testing [20].

In this study, we first developed a screening method based on rapeseed seedlings with grouped compounds from a plant metabolite library. The relatively efficient and affordable screening of 235 groups (1,047 plant metabolites) identified a derivative of paclitaxel, 7‐epi‐10‐deacetyltaxol (designated as L2), with inhibitory effects on cell growth and division in various plant parts that elongate, such as the root, hypocotyl, petiole, inflorescence axis, and placenta, and in different plant species, including dicotyledons (Arabidopsis, rapeseed, and tobacco) and a monocotyledon (rice). We also treated seedlings, adult plants, PSB‐D cell line, and fast‐dividing tissue, or a special callus, namely *Nicotiana tabacum* Bright Yellow‐2 (BY‐2) cell culture, which is used as the “HeLa” cell in cell biology‐related research of higher plants [21]. We further explored the mode of action of L2 in plants by transcriptomics, proteomics, and cytological observation. Similar to animal, plant cell division is inhibited through microtubule stabilization under treatment. However, we also found brassinosteroid (BR) signaling inhibited after L2 ingestion. A comparison between L2 and other antineoplastic drugs showed that L2 has stronger inhibitory effects on the growth of animal cells and various tumor organoids. These findings investigated L2 as a novel growth regulator with broad‐spectrum inhibitory effects on plant and animal cell division, indicating a new screening method and providing the action mode of paclitaxel and derivatives as a reference for potential clinical therapy. More importantly, L2 provided a new sight for understanding how “harmful” metabolites would function for plants.

## 2 Results

### 2.1 A Plant‐Based Metabolites Screening System

**FIGURE 1:**
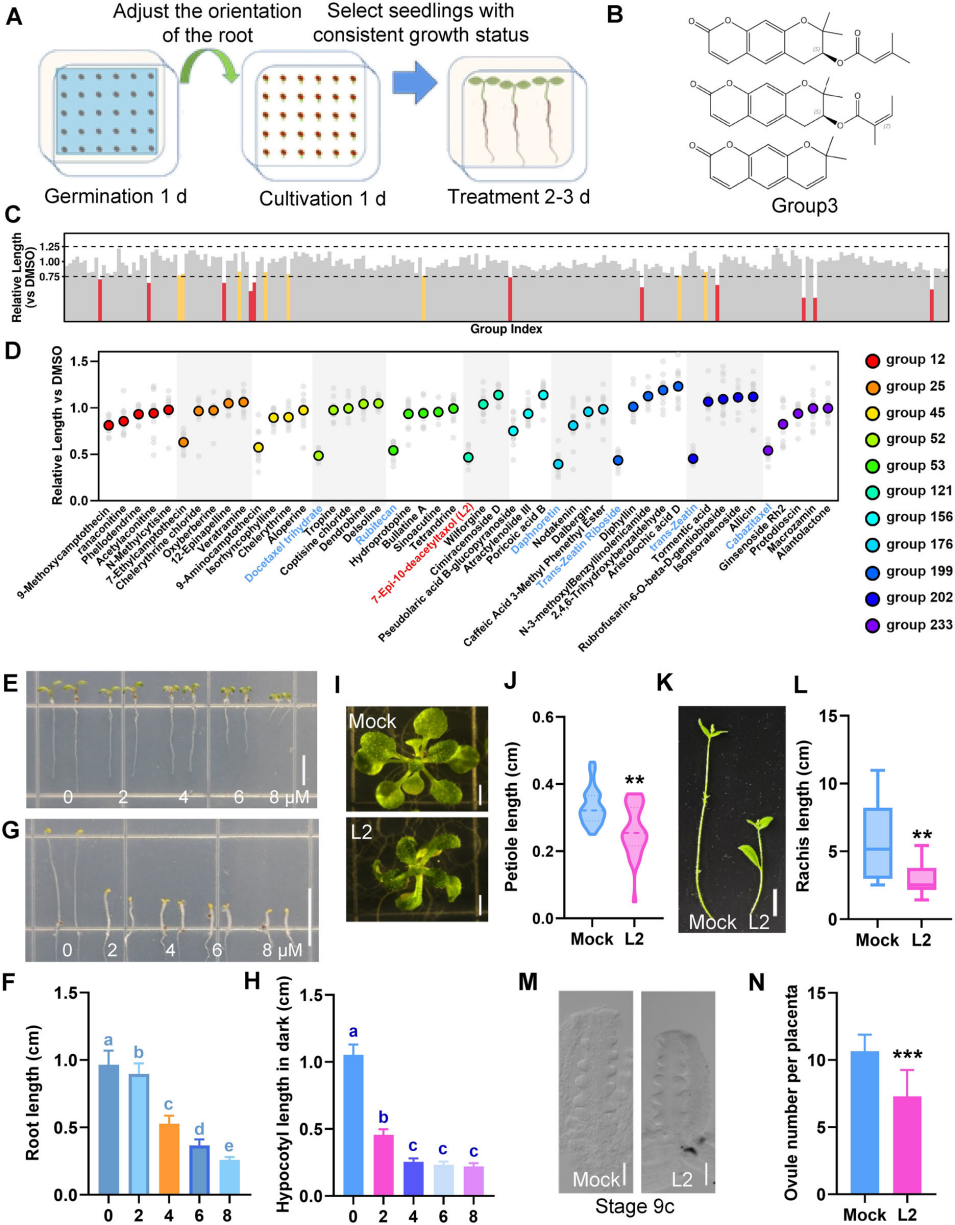
L2 inhibits axial growth in Arabidopsis. (A) Workflow of chemical screening using a plant‐derived compound library in rapeseed Zhongshuang 11. (B) Chemical structures of compounds in group 3 as an example for the grouping principle. (C) Results of grouped screening. The height of each column represents the relative root length of the respective group compared with the mock group treated with DMSO. All colored columns indicate compound groups that effectively inhibited root growth, as judged by visual inspection, and red columns indicate groups reduced by at least 25% through actual measurement. (*n* = 12) (D) Results of treatments with every single compound from the effective groups (red columns in C). Colored points represent the relative length, and blurred points indicate the measured values. Reported drugs or phytohormones are highlighted in blue, and compound 7 epi‐10‐deacetyltaxol for further investigation is highlighted in red. (E) Representative phenotypes of wild‐type seedlings at 5 DAG grown under light and treated with L2 (0, 2, 4, 6, and 8 µm). Bar = 0.5 cm. (F) Quantification of root length in E, *n* > 15. (G) Representative phenotypes of wild‐type seedlings at 5 DAG grown in darkness and treated with L2 (0, 2, 4, 6, and 8 µm). Bar = 0.5cm. (H) Quantification of hypocotyl length in G, *n* > 15. I. Petiole phenotype of rosette leaves. Bar = 2 cm. (J) Quantification of petiole length in I, *n* > 30. K. Inflorescence axis phenotype after continuous application of L2 to the SAM for 2 weeks. (L) Quantification of inflorescence axis length in K, n > 15. (M) Pistil and placenta phenotypes after L2 treatment for 24 h. (N) Quantification of ovule number per placenta in M, *n* > 15. Data are means ± SD. Results are representative of three independent experiments. Statistical significance was assessed by Student's *t*‐test (J, L, N), ^**^ *p* < 0.01, ^***^ *p* < 0.001; or by one‐way ANOVA followed by Tukey's post‐hoc test (F, H), where different letters indicate significant differences (*p* < 0.05).

To explore the effects of plant metabolites on plant cell division and search for undiscovered plant growth regulators (PGRs) or potential anti‐tumor drugs, we analyzed a plant secondary metabolites library with a plant screening system based on rapeseed (*Brassica napus*) seedlings for their large seed number, moderate size, and rapid growth rate. For the screening workflow, rapeseed seedlings with consistent primary root length were transferred to a culture dish with a compound mixture at 2 days after germination (DAG). The root length was then compared at 4 or 5 DAG to examine the effects of the compound mixture (Figure 1A). We first divided 1,047 compounds in the library into 235 groups according to the absence of chemical reactions within the group and, if possible, similarity of the backbone structure (Figure 1B).

For the results of grouped screening, direct visual inspection identified 19 groups, while statistical analysis confirmed 11 groups with a threshold of plus or minus 25 percentage (Figure 1C,D). The 11 groups exerted significant influences on root length, mostly inhibiting root growth, and were analyzed individually, resulting in 9 single compounds presenting effects on root growth. These compounds included reported phytohormones and drugs (e.g., trans‐zeatin and topotecan) [22], demonstrating the validity of our screening method. Based on the extent and consistency of the suppression effect and the number of existing research studies for each single compound, we chose 7‐epi‐10‐deacetyltaxol (L2) for further exploration. We later tested 6 other FDA‐approved anti‐tumor drugs and found 3 of them exhibited inhibitory effects on rapeseed root growth (Figure S1).

### 2.2 7‐Epi‐10‐Deacetyltaxol Significantly Inhibits Plant Growth

We next explored the inhibitory and dosage effects of L2 with Arabidopsis seedlings and found that L2 significantly inhibited Arabidopsis seedling growth under light and exhibited an explicit dosage effect, with 4 µm revealed as a clear inflection point (Figure 1E,F). To assess whether light affected the inhibitory effects, seedlings were treated in darkness, and hypocotyl elongation was inhibited at 2 µm. This effect was more pronounced at 4 µm (Figure 1G,H). These results indicate that L2 inhibits plant growth with different sensitivity at different concentrations, organs, and conditions of culture.

We proposed that L2 affected other tissues that elongate longitudinally and conducted further treatments on Arabidopsis. The lengths of the rosette leaf petiole, inflorescence axis, and pistil decreased significantly after L2 treatment (Figure 1I–N), preliminarily verifying the hypothesis. Arabidopsis has a linear placenta, the length of the placenta directly affects ovule primordia identity and ovule number [23], which is an important seed yield‐related trait. We found that after L2 treatment, the ovule number per placenta decreased by 32% at flower developmental stage 9 (ovule initiation starts) (Figure 1M,N). These results suggest that L2 inhibits longitudinal tissue growth in Arabidopsis.

### 2.3 L2 Inhibits Cell Elongation and Division

The inhibition of longitudinal elongation could be caused by decreased cell division or/and cell elongation. To distinguish the effect of L2, we examined the morphology of Arabidopsis hypocotyl epidermal cells under treatment. Mock control cells exhibited an elliptical shape, whereas L2 treatment resulted in relatively round and short cells (Figure S2A,B), indicating a decreased cell aspect ratio and inhibited cell elongation. L2 also affected root development by inducing blunt root tips and a shorter division zone. Specifically, L2‐treated root crown column cells (columella) were shorter and rounder, and the epidermal cell number in the meristematic zone was significantly reduced (Figure S2C,D). In addition, the epidermal cells of the root elongation zone also exhibited decreased cell aspect ratio (Figure S2E,F). Additionally, L2 induced a reduction in the size of the quiescent center (QC) region, and this effect was further confirmed by the QC‐specific marker line *pWOX5::H2B‐GFP* (Figure S2G,H). These findings suggest that L2 may inhibit cell elongation.

**FIGURE 2:**
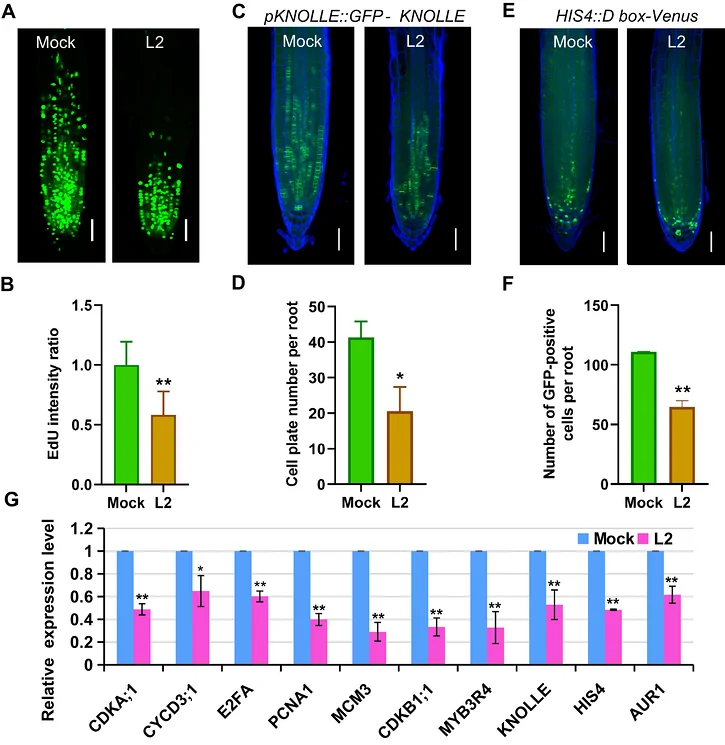
L2 suppresses cell division in Arabidopsis roots. (A) EdU staining of proliferating cells in root tips at 3 DAG. Bar = 50 µm. (B) Quantification of EdU incorporation (intensity ratio). (C,E) Representative fluorescence images of root tips at 5 DAG expressing the cytokinesis marker pKNOLLE::GFP‐KNOLLE (C) and the G1/S marker HIS4::D box‐Venus (E) lines. Cell outlines were counterstained with Fluorescent Brightener 28 (FB28). Bar = 50 µm. (D,F) Quantification of pKNOLLE::GFP‐KNOLLE (D) and HIS4::D box‐Venus (F) fluorescence intensity. (G) Relative transcript levels of cell‐division/cell‐cycle–associated genes including CDKA;1, CYCD3;1, E2FA, PCNA1, MCM3, CDKB1;1, MYB3R4, KNOLLE, HIS4 and AUR1 in roots at 5 DAG. Data are presented as means ± SD for (B, D, F) or means ± SE for (G) from three independent biological replicates (*n* = 3). For imaging quantification in (B,D,F), at least 5, 6, and 8 roots were analyzed per treatment in each biological replicate, respectively. Statistical significance between treatments was assessed by a two‐tailed Student's *t*‐test; ^*^ *p* < 0.05, ^**^ *p* < 0.01.

To elucidate the effect of L2 on cell division, we conducted 5‐ethynyl‐2′‐deoxyuridine (EdU) staining on the roots of Arabidopsis seedlings at 3 DAG because the meristematic zone is active at that time [24, 25], and we observed significantly weaker EdU signals in the L2 treatment group than in the control (Figure 2A,B). The subsequent analysis of the *pKNOLLE::GFP‐KNOLLE* [24, 25], and *HIS4::D box‐Venus* [24] marker lines revealed that the roots of L2‐treated plants had smaller areas with fluorescence signals than the roots of mock control plants (Figure 2C–F). Further detection of cell division‐related genes in the roots of seedlings at 5 DAG showed that the expression levels of cell‐cycle‐related genes all decreased significantly (Figure 2G) [26, 27, 28]. Collectively, L2 tended to impair the cell cycle and morphology, thus inhibiting both cell elongation and division.

### 2.4 L2 Inhibits the Growth of Fast‐Dividing Tissues

**FIGURE 3:**
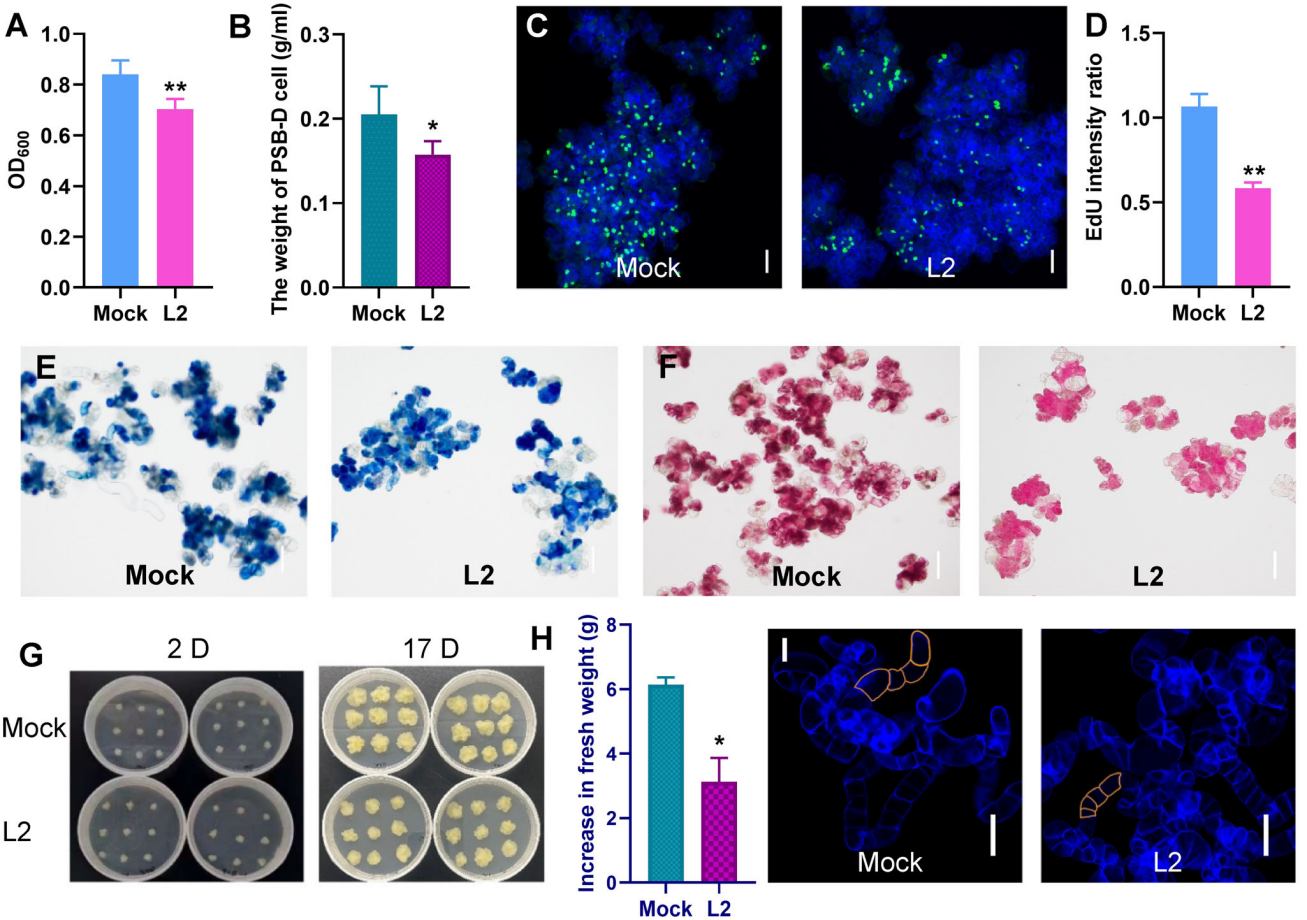
L2 inhibits proliferation of PSB‐D suspension cells and BY‐2 cultures. (A) Optical density of PSB D(MM2d) cultures after 2‐day treatment. (B) Fresh weight of PSB‐D(MM2d) cultures after 7‐day treatment. (C) EdU staining of PSB‐D cells after 1‐day treatment. Cell outlines were stained with FB28. Bar = 100 µm. (D) Quantification of EdU incorporation (intensity ratio). (E) Evans blue staining of PSB‐D suspension cells after 4 µm L2 treatment. Bar = 100 µm. (F) Neutral red staining of PSB‐D cells. Bar = 100 µm. (G) Representative growth phenotypes of BY‐2 cultures on solid medium after 2‐ and 17‐day treatments. H. Increase in fresh weight of BY‐2 cultures after 21‐day treatment. (I) Representative BY‐2 cell morphology with FB28‐stained cell outlines after L2 treatment, four cells are traced for visualization. Bar = 100 µm. Unless otherwise indicated, PSB‐D cultures were treated with 4 µm L2. Data are presented as means ± SE. The sample size (n) represents the number of independent biological replicates (*n* = 5 for A and B; *n* = 3 for D and H). For image quantification in (D), at least 7 cultures were analyzed per treatment in each biological replicate. Statistical significance was assessed by two‐tailed Student's *t*‐test, ^*^ *p* < 0.05, ^**^ *p* < 0.01.

We next investigated whether L2 exerts a similar influence on plant fast‐dividing tissues such as the Arabidopsis PSB‐D cell line and Nicotiana BY‐2 cells. These tissues feature a high rate of cell division, thus are sufficient material for cell division and viability measurement. We examined the effects of L2 on cell proliferation by measuring the absorbance and fresh weight of PSB‐D cells per unit volume. The total biomass and cell density were significantly lower (Figure 3A,B). We employed EdU staining to evaluate the viability of cell division and found fluorescence in large cell clusters was significantly more sparsely distributed in the L2‐treated samples with lower intensity (Figure 3C,D). We also conducted Evans Blue and Neutral Red staining, which respectively indicate cell membrane integrity and vacuole membrane permeability [29, 30]. Compared with the control, PSB‐D cells treated with L2 were more readily stained (Figure 3E), suggesting that the L2 treatment adversely affected PSB‐D cell membrane integrity. Meanwhile, the proportion of PSB‐D cells stained with Neutral Red and staining intensity were significantly lower in the L2‐treated group (Figure 3F). Together, these findings confirmed that L2 markedly inhibited PSB‐D cell division.

We further assessed whether L2 affects BY‐2 cells [41, 42, 52, 53, 54]. After a 17‐day cultivation on media, control BY‐2 cell cultures exhibited robust growth. Conversely, the fresh weight of BY‐2 cells on media containing L2 was markedly suppressed (Figure 3G,H). An analysis of BY‐2 cell morphology detected distinct changes in cell shape and size. Compared with control cells, cells treated with L2 had more irregular shapes (tended to be shorter and rounder) (Figure 3I).

Additionally, we tested L2 when inducing callus from Arabidopsis root cells. The results illustrated that L2 treatment did not significantly affect callus initiation (Figure S3A,D,G). Although microscopic examination along the root length revealed the area of an individual callus increased as the induction period increased (Figure S3B,E,H), the number of calli per unit length was unchanged (Figure S3C,F,I). This result indicated that L2 inhibits cell division rather than cell type conversion. Collectively, these results suggest that L2 possesses the ability to inhibit fast‐dividing tissue growth.

### 2.5 L2 Inhibits Cell Division Through Interfering With BR‐Related Pathway

To elucidate the regulatory mechanism of L2 effects, we conducted a comparative transcriptome sequencing (RNA‐seq) analysis using seedlings grown under light at 5 DAG. The analysis revealed significant differences in gene expression profiles between the L2‐treated and mock control groups (Figure S4A–C). The 30 most significantly enriched Gene Ontology (GO) terms across the three major categories were visualized using bubble plots (Figure S4D). Besides the terms involving cell division, cell wall, and microtubule, which are consistent with our previous findings, we noticed several sets of DEGs within phytohormone‐related pathways (Figures S5 and S6A). Among these phytohormones, auxin, cytokinin, and BR are widely reported to promote plant growth through regulating cell division [27, 31, 32, 33].

**FIGURE 4:**
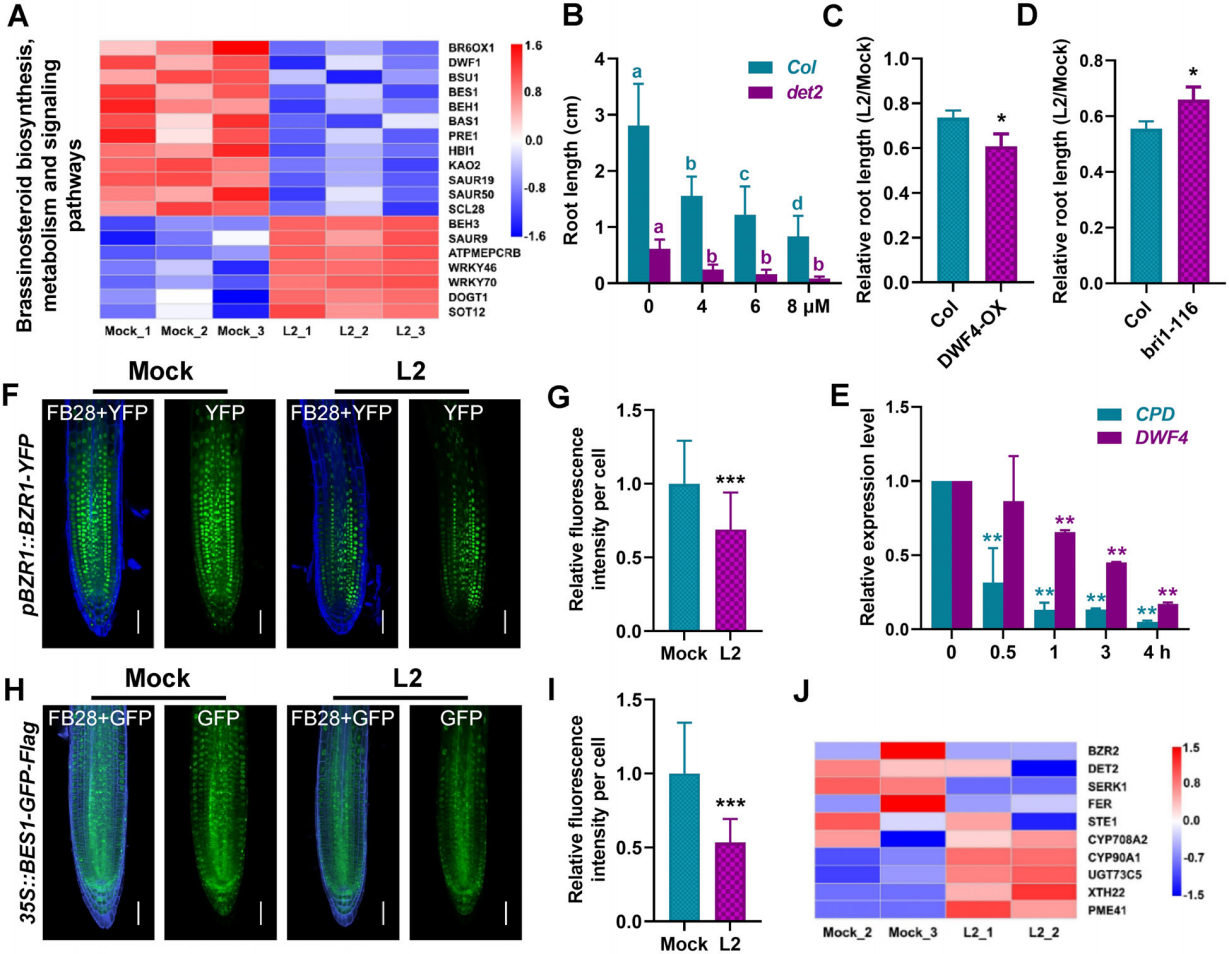
L2 alters BR responses in Arabidopsis. (A) Heat map of 19 BR biosynthesis, metabolism, and signaling‐related DEGs. Red and blue indicate up‐ and downregulation, respectively. (B) Primary root length of Col and det2 seedlings at 12 DAG treated with L2 (0, 4, 6, and 8 µm), *n* > 24. (C, D) Relative root length (L2/Mock ratio) of Col vs. DWF4‐OX (C) and Col vs. bri1‐116 (D) in seedlings at 7 DAG treated with 4 µm L2, *n* = 3 biological replicates. (E) Relative transcript levels of CPD and DWF4 in wild‐type seedlings at 5 DAG treated with 10 µm L2 for 0, 0.5, 1, 3, and 4 h, *n* = 3 biological replicates. (F, H) Representative BZR1‐YFP (F) and BES1‐GFP (H) fluorescence in roots at 5 DAG. Cell outlines were counterstained with FB28. Bar = 50 µm. (G) Quantification of BZR1‐YFP fluorescence intensity per cell in epidermal cells of the meristematic zone, *n* > 74 cells from 8 seedlings. (I) Quantification of BES1‐GFP fluorescence intensity per cell in epidermal cells of the meristematic zone, *n* > 56 cells from 6 seedlings. (J) Heat map of 10 proteins related to brassinosteroid biosynthesis, metabolism, and signaling pathways identified by proteomic analysis. Data in (B, C, D, G, I) are means ± SD or means ± SE (E), from three independent biological replicates. Statistical significance was assessed by two‐way ANOVA followed by Tukey's multiple comparison test (B), two‐tailed ratio paired *t*‐test (C, D), or Student's *t*‐test (E, G, I); different letters indicate significant differences (*p* < 0.05), ^*^ *p* < 0.05, ^**^ *p* < 0.01, ^***^ *p* < 0.001.

Heatmap analysis revealed that both auxin‐ and cytokinin‐related genes were differentially expressed after L2 treatment (Figure S6B,C). Notably, the affected auxin‐related genes included components associated with auxin response, transport, biosynthesis, and developmental regulation, whereas the cytokinin‐related genes were linked to cytokinin biosynthesis/activation, transport, phosphorelay signaling, negative regulation, and downstream responses. However, the transcriptional changes in both hormone‐related gene sets were mixed rather than unidirectional, with both upregulated and downregulated genes detected within each pathway. We therefore examined whether these transcriptional changes were accompanied by altered auxin or cytokinin responses in roots using the corresponding reporter lines, *DR5::NLS‐GFP* and *TCS::GFP*. L2 treatment did not cause obvious changes in either the fluorescence distribution or signal intensity of these reporters in roots (Figure S6D), suggesting that auxin and cytokinin signaling are unlikely to be the major pathways underlying the growth‐promoting effect of L2. By contrast, a series of essential genes involved in BR biosynthesis and signaling showed coordinated expression changes under L2 treatment (Figure 4A). We then treated BR‐deficient mutant *det2* [34] with gradient concentrations of L2, and found that det2 exhibited insensitivity to L2 treatment (Figure 4B). To further assess the involvement of the BR pathway, we compared the relative root length of BR‐related mutants and transgenic lines after L2 treatment (the suitable concentration from Figure 4B). The root growth of *DWF4‐OX*, which is an over‐expression line of a key BR biosynthesis gene, was significantly inhibited than that of *Col* (Figure 4C). Conversely, the root growth of *bri1‐116*, a BR‐insensitive mutant (loss‐of‐function mutant of BR receptor), was less inhibited than that of Col (Figure 4D), indicating that L2 more effectively inhibits root growth in BR‐signaling‐enhanced plant than BR‐signaling‐decreased plant. Based on above phenomenon, we suspect that L2 inhibits root growth partially through inhibiting BR signaling. In BR deficient or insensitive mutants, L2 cannot further decrease BR signal. Thus, BR‐signaling‐decreased mutants are insensitive to L2, suggesting that the intact BR pathway is required for the full growth‐inhibitory response to L2. Besides, the expression levels of important BR biosynthesis genes *CPD* and *DWF4* were lowered, indicating that L2 might also result in a reduced BR level (Figure 4E). We also examined the intensity and nuclear localization of BR‐induced core transcription factors BZR1 and BES1, which reflect BR signaling. In *pBZR1::BZR1‐YFP* and *35S::BES1‐GFP‐Flag* lines, L2 treatment caused a remarkable decrease in YFP fluorescence intensity, particularly in the root (Figure 4F–I). We tested this hypothesis with a differential proteomic analysis of Arabidopsis at 5 DAG. Consistent with the results of transcriptome and RT‐PCR, protein levels of BR biosynthesis and signaling were disturbed (Figure 4J, Figure S7).

Considering that the main downstream genes of BR that promote cell division, *CYCD3;1* and *KNOLLE* [27, 33, 35], were suppressed after L2 treatment (Figure 2C,D,G). L2 likely affects cell division and elongation by interfering with the BR signaling pathway.

### 2.6 L2 and Paclitaxel Differentially Inhibit Cell Division and Tissue Growth in Different Plants

L2 is a derivative of paclitaxel, a natural secondary metabolite derived from the bark of the Chinese yew (*Taxus wallichiana* var. *chinensis* (Pilger) Florin), which has been reported to have significant anti‐tumor effects [36]. It concerns us whether paclitaxel exerts similar effects on plant growth. We conducted treatments on Arabidopsis seedlings grown under light with different concentrations of paclitaxel. Paclitaxel illustrated the obvious inhibition of root growth and hypocotyl elongation with a dosage effect. Similarly, seedlings under treatment in darkness indicated that hypocotyl elongation was suppressed by paclitaxel in a concentration‐dependent manner between 2 and 4 µm (Figure S8A–D). These findings suggest that paclitaxel regulates plant cell division, which is consistent with L2.

To compare the inhibitory effects of paclitaxel and L2 on plant growth, Arabidopsis seedlings were treated with 4 µm paclitaxel or L2. Both L2 and paclitaxel inhibited root elongation, while the inhibition was greater for L2 than for paclitaxel under light (Figure S9A,B). However, there was no significant difference between L2 and paclitaxel in inhibiting hypocotyl elongation in darkness (Figure S9C,D). The inhibitory effects of L2 and paclitaxel on cytokinesis were also evaluated via EdU staining, which revealed greater fluorescent decreases for the L2‐treated samples (Figure S9E,F). Accordingly, L2 appears to inhibit Arabidopsis root elongation and cell division more effectively than paclitaxel.

We next expanded our scope to plants other than Cruciferae. Paclitaxel inhibited rice root elongation significantly more than L2 (Figure S10A,B). Neither compound affected the growth of the above‐ground parts of rice plants (Figure S10C). Tobacco was also treated with both compounds, resulting in inhibited root elongation, but with no difference between L2 and paclitaxel (Figure S10D,E). Collectively, these findings suggest that L2 and paclitaxel have similar effects, but the extent of the effects varies between different plants.

### 2.7 Microtubule Morphology was Altered Upon L2 and BR Treatment

As a paclitaxel derivative [37], L2 likely functions as a microtubule stabilizer, and BR is known to regulate microtubule organization [38, 39, 40]; we therefore investigated how L2 and BR each affect microtubules and how they act in combination.

Consistent with the twisted rosette leaf petioles previously observed under L2 treatment (Figure 1I), prolonged treatment of wild type seedlings with L2 or paclitaxel significantly increased β‐tubulin protein levels (Figure S9G) and markedly enhanced visible cortical microtubule fibers in hypocotyl epidermal cells of *pTUB6::TUB6‐mCherry* seedlings (Figure S9H). Quantitative analysis further confirmed comparable increases in cortical microtubule density (Figure S9I), anisotropy (Figure S9J), and similar shifts in angular distribution (Figure S9K). These results establish L2 as a potential microtubule‐stabilizing compound, comparable to paclitaxel.

**FIGURE 5:**
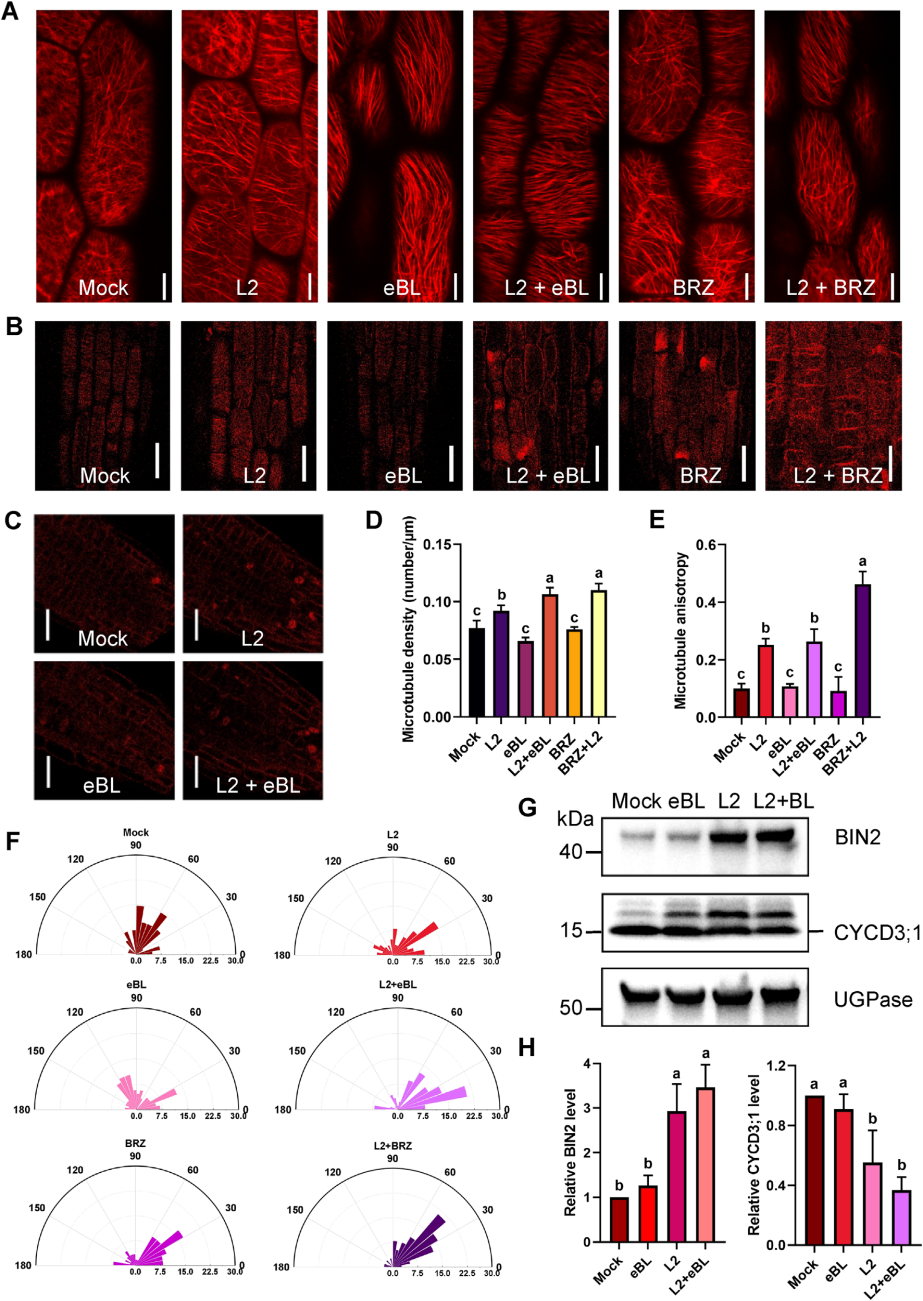
L2 disrupts cortical microtubule organization. (A,B) Representative cortical microtubule arrays in hypocotyl (A) and root (B) epidermal cells of the TUB6‐mCherry line after 6 h treatments as indicated (mock, 10 µm L2, 10 nm eBL, 10 µm L2 + 10 nm eBL, 2 µm BRZ, and 10 µm L2 + 2 µm BRZ, respectively). Bar = 10 µm. (C) Mitotic spindle organization in the largest optical section of the root meristem after 6 h treatment with mock, 10 µm L2, 10 nm eBL, and 10 nm eBL+10 µm L2, respectively. Bar = 25 µm. (D–F) Quantification of microtubule density (D), anisotropy (E), and orientation (F) from (A). For (D) and (E), data are mean ± SD from 3 biological replicates per treatment (≥10 cells per replicate); statistical tests were performed on replicate‐level means. (F) Rose diagrams show the frequency distribution of microtubule orientation angles relative to the cell long axis, measured using FibrilTool and binned into 10° intervals (0–180°); >30 cells per treatment from 3 biological replicates are shown for visualization only. (G) Western blotting of BIN2 and CYCD3;1 in total cell extracts under the indicated treatments (DMSO, DMSO + eBL, L2, and L2 + eBL). UGPase was used as a cytoplasmic loading control. (H) Quantification of BIN2 and CYCD3;1 protein levels in (G), normalized to UGPase and shown relative to mock. Data are mean ± SD from 3 independent biological replicates. For (D,E,H), statistical significance was determined by one‐way ANOVA with Tukey's post hoc test; different letters indicate significant differences (*p* < 0.05).

Because L2 treatment also disturbed BR signaling (Figure 4), we next examined the combined effects of L2 and BR on microtubules using a shorter treatment window that captures the dynamic intermediate state, where the effects of L2 and BR still actively balance each other–a state particularly informative for revealing their distinct contributions. We treated *pTUB6::TUB6‐mCherry* seedlings with L2, eBL, BRZ, L2+eBL, or L2+BRZ, and examined cortical microtubule arrays in hypocotyl epidermal cells (Figure 5A). Two patterns emerged: (i) microtubule abundance was markedly increased by L2, L2+eBL, and L2+BRZ (highest in L2+BRZ), but not by eBL or BRZ alone; (ii) microtubule orientation was transverse in eBL‐ and L2+eBL‐treated cells, but distinctly oblique in L2+BRZ‐treated cells. Thus, L2 and BR appeared to act on different aspects of the cortical array–L2 on abundance, BR on orientation.

To objectively confirm this dichotomy, we quantified cortical microtubule density, anisotropy, and angular orientation using the ImageJ FibrilTool plugin, as previously described [41, 42] (Figure 5D–F). These three parameters are conceptually independent (how many, how ordered, and which direction), together providing a complete description of the array.

Density (Figure 5D) was L2‐dominated. Consistent with the visual observations, L2 alone significantly increased density compared with mock, whereas eBL or BRZ alone did not. Both L2+eBL and L2+BRZ further increased density beyond that of L2 alone, with L2+BRZ reaching the highest value–consistent with a model in which endogenous BR normally “loosens” microtubules (e.g., via suppression of stabilizers such as MBAPs/BIN2 [38, 39, 40]), so that blocking BR biosynthesis by BRZ removes this counteracting force and amplifies L2‐induced effects. Anisotropy (Figure 5E) followed the same L2‐dominated pattern: L2 and L2+eBL significantly increased array ordering, while eBL or BRZ alone had no significant effect. The L2+BRZ group displayed the highest anisotropy value, indicating that the dense cortical microtubule array under this treatment was in fact highly aligned. Notably, anisotropy measures parallel alignment irrespective of direction, so high anisotropy does not imply a productive transverse orientation–an issue resolved by the angular analysis below. In contrast, angular distribution (Figure 5F) was BR‐dominated. eBL‐ and L2+eBL‐ treated cells showed transverse‐enriched orientations, whereas L2+BRZ was enriched at oblique angles. Together, the three parameters delineate a clear division of labor: L2 controls how many microtubules are present and how ordered they are, whereas BR controls which direction they point. The L2+BRZ condition–densest, most aligned, yet locked in an oblique orientation–illustrates that “many and ordered” does not equate to “correctly directed.” According to previous reports, such hyper‐stabilized and obliquely oriented cortical arrays tend to compromise anisotropic expansion and cell division rather than promote them [38].

We next analyzed tubulin fluorescence intensity in the roots (Figure 5B and Videos S1–S6). Consistent with publication [40], tubulin fluorescence intensity was slightly decreased in eBL‐treated but increased in BRZ‐treated roots, indicating that BR status modulates tubulin signal accumulation in root epidermal cells. All L2‐containing treatments showed enhanced fluorescence, with L2+BRZ showing a stronger signal than the L2+eBL group (Figure 5B), mirroring the hypocotyl findings that L2 dominates tubulin accumulation while BR modifies but does not override this effect.

Because the mitotic spindle is built from microtubules and requires dynamic assembly/disassembly, we asked whether L2‐induced altered microtubule architecture also affects the spindle. We analyzed spindles in root endodermal cells after the indicated treatments and found that neither L2 treatment nor BR obviously altered spindle morphology; however, spindle‐containing cells were rarely observed in mock‐ and eBL‐treated roots but were markedly increased after L2 and L2+eBL treatments (Figure 5C).

Together, these results reveal a functional dichotomy: L2 controls microtubule abundance and ordering (Figure 5A,B,D,E), while BR controls orientation (Figure 5F). BR modulates but does not override L2. BRZ even amplifies L2‐induced effects by removing the BR‐mediated “loosening,” producing a densely aligned but misdirected array. Similar excessive alterations extend to spindle microtubules, which is consistent with delayed mitotic progression (Figure 5C). Together, these results provided a cytoskeletal explanation for L2‐induced inhibition of cell division.

### 2.8 L2 has Preliminary Cytotoxicity and Inhibits the Growth of Animal Cells and the Organoid of Ovarian Cancer

Given the varying inhibitory effects of L2 on the growth of plant fast‐dividing tissues, we investigated whether L2 also inhibits the growth of animal cells. We used L2 to treat rat vascular smooth muscle cells. Analyses of videos of live cell dynamics captured by a high‐throughput live‐cell analysis system revealed that the proportion of round cells increased as the L2 concentration increased. Similar results were obtained following treatments with high paclitaxel concentrations (Figure S11A). Cells treated with L2 and paclitaxel migrated more slowly than DMSO‐treated cells (Figure S11B). This observation was validated by statistical analyses of the average migration distance and velocity (Figure S11C,D). Additionally, compared with the 10 µm paclitaxel treatment, the 5 µm L2 treatment had a stronger inhibitory effect on cell migration (Figure S11C,D). To qualitatively assess cell morphological changes, colored polygons were used as cell masks, which revealed that cells became larger and rounder after 24 h of L2 and paclitaxel treatments (Figure S11E). Moreover, the number of cells decreased following treatments with high L2 concentrations (Figure S11F). We hypothesize that this decrease may be due to the weakening of cytoskeletal constraints and the inhibition of cell proliferation caused by high L2 concentrations.

Considering the inhibitory effects of L2 on rat cell growth and division, we further evaluated the effect of L2 on human tumor organoids, including ovarian cancer organoids, intrahepatic cholangiocarcinoma organoids, glioma organoids, and intrahepatic cellular carcinoma organoids (Figure S12A). Three out of four ovarian cancer organoids showed growth inhibition at 5 days post‐treatment (Figure S12B). In the control group, free cells proliferated at the edges of organoids, leading to a gradual filling of the edge gaps and a rounding of the entire organoid mass. By contrast, organoids treated with low or high L2 concentrations maintained distinct edge gaps and smooth edges, with minimal free cell growth. The growth of four intrahepatic cholangiocarcinoma organoids was inhibited by L2, with the most pronounced effect observed for LV134 (Figure S12C). Similarly, the growth of one (BR104) of three glioma organoids was inhibited (Figure S12D). However, the growth of five intrahepatic cellular carcinoma organoids was not significantly affected by any of the tested L2 concentrations at 5 days post‐treatment. These findings suggest that L2 differentially inhibits the growth of various fast‐dividing organoids.

**FIGURE 6:**
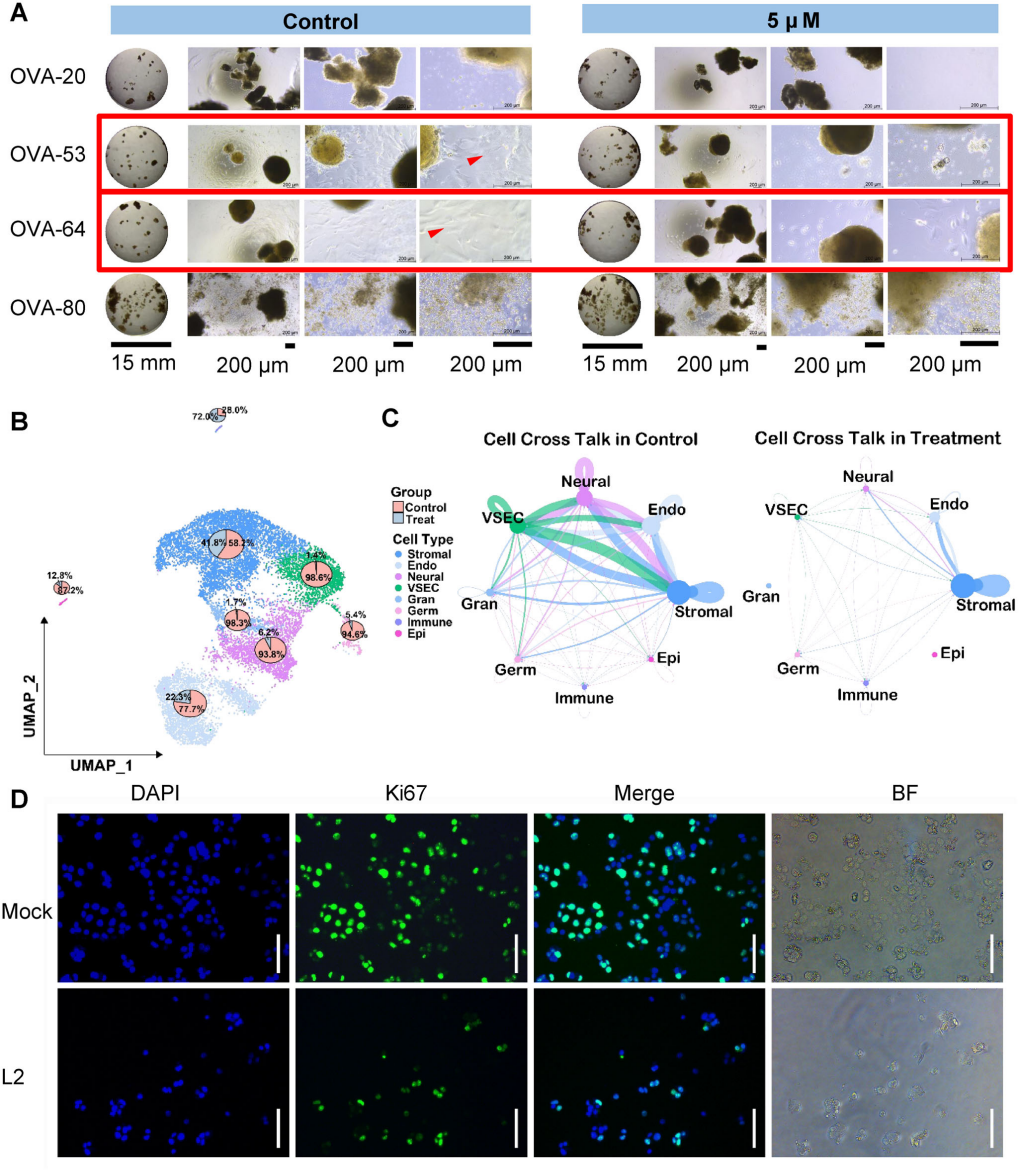
L2 inhibits growth of ovarian cancer organoids and cell line. (A) Representative ovarian cancer organoids after 7‐day treatment with 5 µm L2. Two organoids showing pronounced growth inhibition are boxed. Red arrowheads indicate detached/single cells at organoid edges. The bars are shown below each image column. (B) UMAP visualization of snRNA‐seq data, colored by eight major cell types, each cell cluster includes a pie chart indicating the proportion of control versus L2‐treated cells. (C) Circos plot depicting inferred intercellular communication among clusters in control and L2‐treated groups. (D) Ki67 immunostaining with DAPI nuclear counterstaining in an ovarian cancer cell line after 24 h of treatment with L2. Bar = 100 µm.

On the basis of these observations, we focused on the inhibitory effect of L2 on ovarian cancer‐like organoids and the underlying mechanism. Analyses of the organoids from four new individuals indicated that a 5 µm L2 treatment (high concentration) had inhibitory effects on OVA‐53 and OVA‐64 at 7 days post‐treatment (Figure 6A). One of the samples with distinct inhibition, OVA‐64, was chosen for cryosection. Ki67, a cell proliferation marker, exhibited a decreased immunofluorescence signal in the treatment group (Figure S12E). We infer that L2 affects cell proliferation of ovarian cancer organoids.

### 2.9 SnRNA‐seq Reveals L2 Inhibits Intercellular Communication of Ovarian Cancer Epithelial Cells and Granular Cells

Taxanes have been repeatedly demonstrated to inhibit microtubule depolymerization in animals [43, 44]. However, the effect of L2 on BR signaling in plants reminds us of additional mechanisms through which L2 might act to inhibit animal cell or tissue growth. To clarify this, we conducted a single‐nucleus RNA sequencing (snRNA‐seq) analysis and obtained 12,874 cells, of which 9,790 and 3,084 cells were from the mock control and L2 treatment groups, respectively. We classified eight major cell types based on uniform manifold approximation (UMAP) analysis according to their gene expression. Notably, we identified 5,352 stromal cells with high *ACSL4* expression levels, 2,824 endothelial cells with high *EFNA5* expression levels, 1,838 perivascular cells with high *SORBS1* expression levels, 460 granular cells with high *FGF7* expression levels, 279 germ cells with high *TOP2A* expression levels, and other cells included immune cells, epithelial cells, and neural cells (Figure 6B). Most of the major cell types were substantially altered by L2 treatment (Figure 6B). Further analyses of cell cross‐talk revealed that L2 eliminated cell‐cell communication in epithelial and granular cell populations (Figure 6C), implying that L2 may target epithelial and granular cells to suppress ovarian cancer.

To further characterize the transcriptional shifts underlying L2‐induced growth inhibition, we examined the expression of curated gene sets representing proliferation/viability, apoptosis, and cell‐cycle regulation across all eight identified cell types (Figure S13). Consistent with the loss of cell‐cell communication observed in epithelial and granular cell populations, these two cell types displayed the most prominent transcriptional shifts following L2 treatment. In epithelial cells, L2 treatment was accompanied by decreased expression of *MYC* and several cell‐cycle regulators (*CDK7*, *CDKL1*, *CDKL5*, *CDK5R1*, and *CDKN1C*), together with increased expression of the apoptosis‐related genes *CASP7* and *PERP*. In granular cells, expression of the cell‐cycle inhibitor *CDKN1C* decreased while the pro‐apoptotic gene *CASP7* increased following treatment, indicating a similar shift toward reduced cell‐cycle progression and enhanced apoptotic signaling. In addition, several cell cycle‐related genes, including *CDK1*, *CDK6*, *CDK10*, *CDK14*, and *CDK17*, were also altered by L2 treatment across multiple cell types. Collectively, these results indicate that L2 disrupts the transcriptional balance between proliferation, cell‐cycle progression, and apoptosis in ovarian cancer organoids, particularly within the epithelial and granular cell populations that are most affected at the level of cell‐cell communication.

To further validate the results above, we treated the ovarian cancer cell line NIH:OVCAR‐3 with L2. It was found that the ovarian cancer cell number within the field of view decreased (Figure 6D). In addition, the ratio of the Ki67 fluorescence signal to DAPI‐stained cell nuclei and the signal intensity of some parts also decreased. Collectively, these results suggest that L2 has the ability to inhibit the proliferation of ovarian cancer cells.

## 3 Discussion

### 3.1 Establishment of an Efficient and Cost‐Effective Chemical Screening System

This study assessed axial growth in various plant tissues (root, hypocotyl, petiole, inflorescence axis, and placenta) and found that L2 significantly inhibited growth in both vegetative and reproductive tissues of Arabidopsis (Figure 1E–N). L2 also altered the size and shape of various plant cell types, including root and hypocotyl cells (Figure S2). Staining of PSB‐D cell lines, which reflected cell viability, indicated that L2 changed the cell growth status (Figure 3A,B). Tobacco BY‐2 cell shape and tissue structure also suggested L2 can suppress cell growth in plant fast‐dividing tissues (Figure 3G–I). Collectively, these findings imply L2 inhibits cell growth in whole plants as well as in plant cell lines and fast‐dividing tissues. Notably, L2 suppressed the growth of different plants, including dicots (Arabidopsis, rapeseed, and tobacco) and a monocot (rice) (Figure 1; Figures S1 and S10). Despite the obvious differences among these plants, L2 had significant inhibitory effects on the root growth of all of these seedlings grown under light.

The inhibitory effects of L2 on cell growth were further demonstrated by treatments of various animal materials, including rat vascular smooth muscle cells and human tumor organoids, three‐dimensional structures that closely replicate the architecture and function of human organs, thus providing a more realistic environment for testing drug efficacy and toxicity (Figures S11 and S12). The animal primary cells used in this study died after 6–8 generations, thereby mimicking the cell growth status in vivo, unlike cell lines that can proliferate indefinitely. The shape and migration of vascular smooth muscle cells indicated that L2 influences cell viability and affects the cell growth status (Figure S11). Moreover, the observed decrease in cell outgrowth reflected the inhibitory effect of L2 on tumor organoid cell growth (Figure S12B–D). The abnormal gene expression and cell‐cell communication revealed by the snRNA‐seq analysis of ovarian cancer organoids showed that L2 suppresses fast‐dividing tissue growth (Figure 6B,C).

Animals and plants share common regulatory mechanisms for cell division [5, 45]. Therefore, plant systems may be useful for screening anti‐tumor drugs. Traditional screening systems for drug discovery are typically divided into two classes: target‐based drug discovery (TDD) and phenotypic drug discovery (PDD). The plant‐based screening system developed in this work appears to be one of PDD systems by definition. However, considering the fact that the system was built on certain mechanisms identical across plants and animals such as cytoskeleton and topoisomerase, our system also has some features of mechanism‐informed phenotypic drug discovery (MIPDD), which is categorized by conducting phenotypic assays for specific targets [46]. Arabidopsis or rapeseed seedlings grow rapidly, with roots and hypocotyls elongating significantly within a few days and thus can serve as indicators of cell division.

Notably, 6 FDA‐approved drugs were also tested, and only 3 of them worked in the system: paclitaxel, docetaxel, and artemisinin. Interestingly, they are all plant metabolites or derivatives. Among the rest 3 compounds, pirarubicin and altretamine, respectively, inhibit the activity of human topoisomerase II and dihydrofolate reductase, which differ massively in plants. Temozolomide targets tumor cells characterized by low levels of O6‐alkylguanine DNA alkyltransferase (OGAT), which is absent in most plants [47]. This explains why not all anti‐cancer drugs function in our screening system.

### 3.2 7‐epi‐10‐Deacetyltaxol Inhibits Plant Tissue/Organ Elongation and Growth Through Multiple Intersecting Pathways, Including BR and Microtubules

After confirming the inhibitory effects during treatments of different plants and plant cell lines, we sought the concrete mechanism within by transcriptomics and proteomics. We found plenty of phytohormone‐related genes altered in the amount of both transcripts and proteins. Phytohormones are known for their all‐embracing effects on plant growth and reproduction, and it is thus rational that the inhibitory effects of L2 go through certain phytohormone pathways. Given the results that L2 inhibits both cell division and elongation, we focused on auxin, BR, and CK because they were reported to be responsible for these aspects and were also found to be altered in the results of omics. We treated marker lines of vital components of these pathways such as *DR5::NLS‐GFP*, *TCS::GFP*, *pBZR1::BZR1‐YFP*, and *35S::BES1‐GFP‐Flag* with L2. Significant changes were only observed in *pBZR1::BZR1‐YFP* and *35S::BES1‐GFP‐Flag* (Figure 4F–I, Figure S6D). Together with the results that a set of core BR biosynthesis and receptor genes and transcription factors, including CPD, BRI1, and BES1, were down‐regulated, the low levels of *CPD* and *DWF4* may indicate either low BR signaling resulting from low BR synthesis or negative feedback due to high BR signal. Considering other results, we believe this reflects low BR synthesis signaling. We conclude BR‐related pathways were impaired under L2 treatment. This explains the restricted elongation. The current study demonstrated that BR activity fluctuates throughout the cell cycle, especially increases during the G1 phase, which induces *CYCD3;1* gene expression and cell division [33]. L2 treatment decreases the intensity of BZR1 and BES1 (Figure 4F–I), as well as the transcription of *CYCD3;1* (Figure 2G), suggesting L2 possibly inhibits cell division through a BR‐related way.

Paclitaxel was well‐studied in animals for its ability to stabilize tubulin and anti‐tumor effects. However, few studies have demonstrated whether a similar effect exists in plants. We tested paclitaxel, L2, and docetaxel, another derivative reported as a stronger anti‐tumor agent, on rapeseed and observed growth inhibition. We conclude the derivatives of paclitaxel possess a similar ability to combine with tubulin and examined the microtubule dynamics in paclitaxel‐ and L2‐treated roots. We observed increased and morphologically altered microtubules in L2 and paclitaxel‐treated groups than non‐treated group, with L2 stronger than paclitaxel. Notably, we found the spindle apparatus in L2‐treated roots more than non‐treated roots, which implies cell division was delayed or blocked. This partially explains the inhibited cell division. On the other hand, BR was also reported to regulate the stability of microtubules through a BIN2‐relevant approach, which corresponds to the results that BIN2 was up‐regulated under treatment [39] (Figure 5G,H). We further explored the additive effects of BR and L2. They appeared to contribute to the stability of microtubules in different ways. As reported, a high BR level leads to lower microtubule stability, and it is rational that a low BR level leads to higher stability. However, despite the similar amount of tubulin, the microtubule morphology under low BR level did not ensemble that under L2 treatment on the polymerization of tubulin. This implies L2 does not impact microtubules through BR. In other words, L2 inhibits plant growth through both BR and microtubule pathways.

According to previous publications [38, 40], microtubules act as downstream targets of BR signaling through the BZR1‐MDP40 module, while microtubule status can in turn feedback on BR signal transduction. Thus, it is important to clarify the causal relationship between BR and microtubules upon L2 treatment to uncover the linkage connecting L2 and BR. Based on our results, we suspect that L2 is more likely to regulate cell division and root growth by separately affecting BR and microtubules, rather than regulating root growth by BR‐mediated changes in microtubule organization, or by microtube‐mediated changes in BR signaling. If L2 suppresses BR signaling by disrupting microtubule organization and thereby inhibits root growth, the BR‐signaling‐enhanced plant, *DWF4‐OX*, would be insensitive to L2 because its elevated endogenous BR level would buffer the L2‐imposed reduction; however, *DWF4‐OX* instead displayed enhanced sensitivity (Figure 4C). Meanwhile, the BR‐deficient or BR‐insensitive mutants *det2* and *bri1‐116* showed reduced sensitivity to L2 (Figure 4B,D), and together with *DWF4‐OX* they define a positive relationship between endogenous BR level and L2 sensitivity, a trend opposite to what a microtubule‐primary model would predict. These observations support that L2 interferes with BR‐related signaling and microtubules at the same time.

### 3.3 The Mode of Action of paclitaxel in Plants Could Act as a Reference for Its Mode of Action in Humans

We believe studying how paclitaxel or L2 works in plants should provide insights into their therapeutic use. We mentioned that plant fast‐dividing tissues were treated to explore how L2 works. Notably, we noticed that despite the intense inhibitory effects on growth of PSB‐D and BY‐2 cells, it appeared that L2 did not impact the formation of Arabidopsis callus (Figure 3, Figure S3). Although callus ensembles plant stem cells than cancer cells, the result still indicated that L2 has the potential to precisely inhibit cell division without any impact on cell identity transformation. We conclude that L2 could work jointly with the therapy aiming to turn cancer cells back to normal cells.

As a plant steroid hormone, BR has a similar structure to some animal hormones (estrone, testosterone, etc.), which might be a reasonable explanation that L2 is easily identified by the plant screening system. However, there are only a few studies about functional comparison of plant and animal steroid hormones [48], which is worth investigating in the future. Our results revealed that L2 inhibits plant cell division possibly through a BR‐related way (Figure 4). A previous report indicated estrogen promotes cell proliferation in epithelial ovarian cancer and breast cancer cells MCF‐7 (by inducing cyclin G1 expression and affecting cell cycle) [49, 50, 51]. There is structural similarity between plant brassinosteroids and animal estrogens, suggesting that the molecular mechanism of L2 inhibiting cell proliferation and fast‐dividing tissue growth in animals might also be via steroid‐relevant ways.

To date, a considerable amount of work has focused on the effects of paclitaxel on cell signaling other than microtubule [52, 53, 54]. This is aligned with our results that L2 inhibits both BR signaling and microtubule depolymerization. Combined with the fact that hormones also play a certain role in cancerization. This wonders us how paclitaxel or L2 would cooperate with hormonal drugs.

### 3.4 Therapeutic Potential of L2 as a Substitute for Paclitaxel

Paclitaxel is a well‐known efficient anti‐tumor drug [37]. We compared the effects of L2 and paclitaxel as well as other candidate molecules. Results suggest L2 may inhibit fast‐dividing tissue growth. Although both L2 and paclitaxel suppressed cell division and cell growth, their effects varied across species and tissues (Figures S1, S9–S11 and S13). Notably, in primary rat vascular smooth muscle cells, low L2 concentrations were more effective than high paclitaxel concentrations (Figure S11). Moreover, L2 had relatively stable effects on Arabidopsis (Figure S9A,B). Thus, L2 may be more efficient or stable than paclitaxel in some cases, warranting further exploration of its potential as an anti‐tumor drug.

Considering that both paclitaxel and L2 exist in Chinese yew, we focused on how L2 is synthesized in vivo and whether there is any overlap with the paclitaxel biosynthetic pathway. Surprisingly, L2 was not associated with the canonical paclitaxel biosynthetic pathway [36], eliminating the possibility that L2 is an intermediate during the synthesis of paclitaxel. Hence, we speculate that L2 formation involves a pathway that branches off the paclitaxel biosynthetic pathway. This speculation is supported by the fact that L2 differs from paclitaxel only in terms of an acetylation at C10 and the stereochemistry of the hydroxyl group at C7. The missing acetylation may be associated with the substrate tolerance of baccatin III‐13‐O‐phenylpropanoyl transferase (BAPT), which can convert 10‐deacetylbaccatin III, instead of baccatin III, directly to 10‐deacetylpaclitaxel without the acetylation catalyzed by 10‐deacetylbaccatin III‐10‐O‐acetyltransferase, resulting in a biosynthetic pathway with one fewer step. However, the difference in stereochemistry is much more difficult to explain. In the canonical paclitaxel biosynthetic pathway, a taxadien‐5α‐13α‐diol precursor undergoes a series of oxidation and cyclization reactions to generate 2‐debenzoyltaxane, with 7β‐hydroxylase (T7βH) introducing a hydroxyl group at C7 [55]. However, existing functional studies on T7βH did not introduce hydroxyl group stereoisomerism. Considering L2 may be another efficient anti‐cancer drug with a shorter biosynthetic pathway, structurally modifying BAPT to increase its selectivity for deacetylated substrates may be conducive to the production of increasingly efficient anti‐cancer agents at relatively low cost.

Several previous studies identified L2 from high‐throughput screening on animal cells and performed limited research on human cancer cell lines and found the inhibitory effects accompanied by likely high cytotoxicity [56, 57]. To evaluate the potential toxicity of L2, we employed a zebrafish embryo model, an increasingly used system for preclinical drug safety assessment [58, 59]. Embryos were continuously exposed to L2 or paclitaxel (10, 20, and 40 µm) in E3 medium from 6 to 72 h post‐fertilization (hpf). Compared to paclitaxel, L2 exhibited lethality at 40 µm and induced developmental abnormalities (Figure S14A). However, we noted significant precipitation of paclitaxel in E3 medium, which may have compromised its observed toxicity. For a more reliable comparison, we tested L2 against the soluble chemotherapeutic agent irinotecan (CPT‐11). At 40 µm, L2 demonstrated comparable lethality to irinotecan but caused milder developmental defects (Figure S14B). Notably, irinotecan induced severe cardiotoxicity, which was not observed with L2 treatment (Figure S14C). These results indicate that while L2 has toxicological properties similar to those of irinotecan, it exhibits a relatively favorable safety profile, indicating promising potential for further development.

### 3.5 Speculations About Why Plants Produce “Harmful” Metabolites

In this work, we reported L2 possesses a highly conserved function of inhibiting cell division in plants and animals. Despite the similar function, the mechanisms of suppression appeared to be different in these organisms. In plants, L2 inhibits cell division and elongation through both BR and microtubule pathways. In animals, L2 interrupted cell communication and also changed cell morphology, probably through the cytoskeleton, like paclitaxel.

The phenomenon of L2's broad inhibition inspired us to its ecological importance to plants. Given the information that taxanes were first extracted from the bark of *Taxus*, where the concentration of taxanes, including paclitaxel and L2, is high enough to exhibit cell toxicity. Thus, when pests or herbivores gnaw the bark, the taxanes would function as defenders. The defense could be extended to bacterial infection under certain circumstances.

In conclusion, we developed a simple, affordable, and efficient large‐scale screening system that identified L2 as an effective small‐molecule compound capable of suppressing cell growth and division in plants (whole plants, plant cell lines, and plant calli) and animals (animal primary cell lines, human cancer organoids, and cell line). L2 has effective and stable effects on cell division in most plants and animals, indicative of the potential utility of L2 as an anti‐tumor drug used to treat specific classes of cancer. More interestingly, the existence of L2 provides a glimpse at the ecological importance of “harmful” plant metabolites such as taxanes.

## 4 Experimental Section

### 4.1 Selection and Grouping of Compound Libraries

The primary screening library was sourced from TOPSCIENCE Corporation. Alkaloids, flavonoids, terpenoids, and coumarins were structurally selected from the Selected Plant‐Sourced Compound Library (L4600). After removal of redundant structures, 1047 unique small‐molecule compounds were retained. Compounds were dissolved in dimethyl sulfoxide (DMSO) to prepare 10 mm stock solutions. For secondary screening, three to five compounds were pooled at equal molar ratios to generate 237 mixtures.

### 4.2 Primary Screening of a Small‐Molecule Compound Library

Seeds of European rapeseed (*Brassica napus*) Zhongshuang 11 (ZS11) [60] were evenly placed on filter paper wetted with distilled water to germinate, and were reoriented vertically at one DAG. For mixture treatments, 10 µL of each mixture stock was added to 6 mL distilled water, and DMSO was used as the mock control. At 2 DAG, 12 seedlings with uniform primary root lengths were transferred to one‐third of a square Petri dish, imaged, and incubated vertically for an additional 2–3 days.

For single‐compound screening, 10 µL of each compound stock was added to 6 mL distilled water (mock: DMSO), and all subsequent steps were identical to those used for mixture screening.

All assays were performed at 22°C, under a 16 h light, and 8 h dark photoperiod.

### 4.3 Small‐Molecule Treatment of Arabidopsis, Rice and Tobacco

Stock solutions of 7‐epi‐10‐deacetyltaxol (L2, T5738, TOPSCIENCE) and paclitaxel (T0968, TOPSCIENCE) were prepared at 10 mm in DMSO. For validation, compounds were added to 10 mL 1/2 MS medium (M519, Phytotech) supplemented with 1% sucrose to final concentrations of 0, 2, 4, 6, and 8 µm (with 0 µm serving as the DMSO mock control)., respectively. Arabidopsis seeds were surface‐sterilized with 75% ethanol for 5 min, and 5% sodium hypochlorite for 5 min, rinsed 3–5 times with sterile distilled water, plated on 1/2 MS medium, vernalized at 4°C for 2 days, and grown vertically for 5 days or 12 days. Stock solutions of 7‐epi‐10‐deacetyltaxol (L2, T5738, TOPSCIENCE) and paclitaxel (T0968, TOPSCIENCE) were prepared at 10 mm in DMSO. For dark growth, plates were wrapped with aluminium foil.

For shoot apical meristem (SAM) assays, 10‐DAG Arabidopsis seedlings grown on 1/2 MS medium were treated with 4 µm L2 for 10 days, respectively. Petiole phenotypes were documented (DFC450, Leica). The inflorescence axis length was measured after continuous dropwise application of a 4 µm compound solution to the SAM at 21‐DAG seedlings for 2 weeks in the greenhouse.

Rice (*Oryza sativa*, ZH11) seeds were germinated for 4 days, then treated with 4 µm compound (mock: equal volume of DMSO). 21 rice seedlings with uniform root lengths were vertically incubated for 4 additional days and imaged.

Tobacco seedling treatments were performed similarly to rice. Briefly, ∼15 tobacco seedlings at 2 DAG were placed on filter paper soaked with 4 µm compound solution, incubated vertically for 9 days, and imaged.

For pistil treatment, inflorescences containing 2–3 florets were collected and peripheral florets were removed. A treatment solution containing 0.01% Silwet L‐77 solution and 4 µm L2 was applied (mock: DMSO). Pistil treatment, fixation and clearing were performed as described previously [61, 62]. Placenta and ovule morphology were examined by DIC microscopy (BX63, OLYMPUS).

Seedlings were grown in a growth chamber and adult plants in a greenhouse, both at 22°C, under a 16 h light / 8 h dark photoperiod.

Root, hypocotyl, and shoot lengths were quantified using ImageJ.

### 4.4 Compounds Treatment of the Arabidopsis PSB‐D Cell Line

Arabidopsis PSB‐D suspension cells were subcultured (1:10) into 30 mL MSMO medium (4.43 g MS, 30 g sucrose, 500 µL NAA, 50 µL kinetin, pH 5.7) after 7–9 days. L2 was added to 4 µm (mock: DMSO). Cultures were maintained at 24°C, with shaking at 150 rpm, protected from light. Samples were collected at one day for EdU staining, and at 2 days for Neutral Red and Evans Blue staining [29], as well as 2 days for absorbance and 7 days for fresh weight measurements.

### 4.5 BY‐2 Cell Culture and Treatment

BY‐2 culture conditions were modified from Yang et al. [25]. L2 was added to BY‐2 medium (PM1591, Coolaber) to a final concentration of 4 µm. Equal fresh weights of BY‐2 cells were divided into nine blocks and transferred to the medium. After 17 days at 28°C and protection from light, total fresh weight per plate was determined. For morphology analysis, BY‐2 cells were gently dispersed into suspension cells in liquid medium and then cultured for 7 days at 28°C, 150 rpm, protected from light.

### 4.6 RNA‐seq Analysis

Arabidopsis *Col* seedlings were grown as described above on 1/2 MS medium supplemented with 4 µm L2 or DMSO as the mock control. At 5 DAG, seedlings were rapidly root‐clipped and immediately frozen in liquid nitrogen.

RNA extraction, library construction, and sequencing were performed by Novogene (Novaseq‐PE150), generating ∼6 G of raw data per sample. Adapter‐containing and low‐quality reads (Qphred ≤ 5 accounting for >50% of read length) were removed using CASAVA. Clean reads were aligned to the TAIR10 genome using HISAT2 v2.0.5, and read counts were obtained with featureCounts (1.5.0‐p3). Gene expression was quantified as FPKM. Differential expression was analyzed using DESeq2 (1.20.0), genes with adjusted *p* ≤0.05 were considered differentially expressed. GO enrichment and KEGG pathway analyses were performed with clusterProfiler (3.8.1), with *p* < 0.05 as the significance threshold.

### 4.7 Quantitative Real‐Time PCR

Roots from 5 DAG seedlings grown under light were rapidly harvested, snap‐frozen in liquid nitrogen, and ground into a fine powder. Total RNA was extracted using TRIzol reagent (15596‐026, Invitrogen), quantified with a spectrophotometer (Nanodrop 1000), and reverse‐transcribed using FastKing RT Kit with gDNase (KR116, Tiangen). qRT‐PCR was performed with ChamQ Universal SYBR qPCR Master Mix (Q711‐02; Vazyme) on a qTOWER3G touch quantitative PCR system (Analytik Jena). Transcript levels of *CDKA;1*, *CYCD3;1*, *E2FA*, *PCNA1*, *MCM3*, *CDKB1;1*, *MYB3R4*, *KNOLLE*, *HIS4* and *AUR1* were calculated using the 2^–ΔΔCT^ method. UBQ10 (AT4G05320) and EF1α (AT5G60390) were used as reference genes [63].

All primers are listed in Table S1.

### 4.8 EdU Staining

EdU staining was modified from Yin et al. [64]. *Col* seedlings at 3 DAG were transferred to liquid 1/2 MS medium containing 10 µm EdU and 4 µm compound and incubated at 22°C for 1 h, fixed in 4% paraformaldehyde fixative, then processed using the EdU detection cocktail (488 Click‐iT EdU Imaging Kit, ShaReBio).

For PSB‐D cells, 1 mL of culture was pelleted by low‐speed centrifugation, resuspended in 1 mL of MSMO medium containing 10 µm EdU and 4 µM L2, and processed as described for seedlings.

Confocal imaging of marker lines and EdU staining: Seeds of pKNOLLE::GFP‐KNOLLE, HIS4::D box‐Venus, DR5::NLS‐GFP, TCS::GFP, pBZR1::BZR1‐YFP, 35S::BES1‐GFP‐Flag and pWOX5::H2B‐GFP were sterilized and grown as described on 1/2 MS medium containing 4 µM L2 (mock: DMSO). Seedlings at 5 DAG were fixed in 4% paraformaldehyde under vacuum for 0.5 h, washed three times with 1× PBS (pH 7.4), and cleared with ClearSee [65].

To visualize cell outlines, cleared seedlings, PSB‐D and BY‐2 cells were stained in fresh ClearSee containing 0.2% Fluorescent Brightener 28 (FB28; F3543, Sigma) for 2 h in the dark. Fluorescence was imaged using a confocal microscope (Ni‐E A1 HD25, Nikon). The excitation wavelength of FB28 was 355 nm, that of GFP, YFP, and EdU was 488 nm, and that of Venus was 485 nm.

For live imaging of cortical microtubules, *TUB6‐mCherry* seedlings were evaluated directly without fixation. Hypocotyl epidermal cells were imaged after 5 days of vertical growth on 1/2 MS medium containing DMSO (mock), 4 µm L2, or 4 µm paclitaxel. Additionally, both hypocotyl and root epidermal cells were imaged after transferring untreated 5‐DAG seedlings for 6 h to media supplemented with DMSO, 10 µm L2, 10 nm eBL, 10 nm eBL + 10 µm L2, 2 µm BRZ, or 2 µm BRZ + 10 µm L2.

The fluorescence intensity of *pBZR1::BZR1‐YFP* and *35S::BES1‐GFP‐Flag* line were quantified with ImageJ.

Cortical microtubule density, anisotropy, and orientation were analyzed using ImageJ and the FibrilTool plugin [41], following the methods described in Liang et al., and Ma et al. [42, 66]. Briefly, density was measured by the line‐intersection method, and anisotropy and average fibril orientation were quantified from polygonal ROIs using FibrilTool. Individual cells were treated as technical subsamples and were not considered independent biological observations.

### 4.9 Western Blotting

Arabidopsis wild‐type seedlings were grown as described above. For microtubule‐stabilization assays, *Col* seeds germinated and grown on 1/2 MS plates containing DMSO (mock), 4 µm L2, or 4 µm paclitaxel for 8 days, and then frozen in liquid nitrogen. For the long‐term L2 combined with short‐term eBL treatment, *Col* seeds were germinated and grown on 1/2 MS plates containing either DMSO or 4 µm L2 for 14 days; the seedlings were then transferred to liquid 1/2 MS medium supplemented with 1 µm eBL or an equal volume of solvent as mock and incubated for 45 min before freezing in liquid nitrogen. Total proteins were extracted, quantified, and subjected to immunoblotting as described previously [62]. Primary antibodies used were anti‐β‐tubulin (SB‐AB0039, Share‐Bio), anti‐BIN2 (PHY3568A, PhytoAB), anti‐CYCD3 (R3493, Abiocode), anti‐UGPase (AS05086, Agrisera) and anti‐Histone H3 (SB‐CY6587, Share‐Bio).

### 4.10 Arabidopsis Callus Induction

*Col* seeds were sterilized as described previously. Callus induction and subculture were performed as Chen [35]. Seedlings at 7 DAG were transferred to callus induction medium (1/2 MS + 0.5 mg/L 2,4‐D + 0.05 mg/L 6‐BA) and cultured at 22°C, under a 16 h light, 8 h dark photoperiod. Clearing and observation were performed as described by Wu [67]. Callus initiation was examined in 50% glycerol using an Axio Imager M2 (ZEISS).

### 4.11 Rat Vascular Smooth Muscle Cells Assays

Primary rat vascular smooth muscle cells were isolated from the aorta of 180 to 200 g male Sprague–Dawley rats using the explant method [68]. Briefly, the aorta was stripped of adventitia and intima, washed with PBS, minced, and cultured in DMEM (Thermo Fisher Scientific) supplemented with 10% FBS (Sigma–Aldrich, St. Louis, MO, USA) and 1% penicillin‐streptomycin. Passage 4–8 cells were seeded in 24‐well plates. Treatments included blank and DMSO controls, L2 (1, 5, 10 µm), and paclitaxel (10 µm). Live‐cell imaging was performed on an Operetta CLS system (PerkinElmer) for 24 h at 30 min intervals. Cell migration was quantified using the TrackMate plugin in ImageJ [69]. Cells numbers at 0 were counted in the field, and 24 h were determined using Cellpose 3.0 (cyto3 model) with manual correction [70], the ratio (*t* = 24 h)/(*t* = 0 h) was used to assess cell proliferation.

### 4.12 Culture and Imaging of Human Cancer Organoids

For the first culture, selected organoids (∼500 µm) were seeded into 96‐well plates (24 spheres/well) in 100 µL carcinoid medium (LSTO015004; Shanghai LiSheng Biotech, China). Organoids were derived from patients with ovarian cancer (*n* = 4) [71, 72], intrahepatic cholangiocarcinoma (*n* = 4) [73], glioma (*n* = 3), and intrahepatic cellular carcinoma (*n* = 5) [73]. For each organoid type, 6 wells were used per group (mock, 1 µM L2, and 5 µm L2, each in duplicate). Plates were incubated at 37°C with 5% CO_2_ and imaged at 0, 1, 3, and 5 days. Images were acquired on day 5 for ovarian cancer organoids and on day 3 for intrahepatic cholangiocarcinoma and glioma organoids (DP70, Olympus).

For the second culture, ovarian cancer organoids from 4 patients were cultured similarly (6 wells per patient, 3 replicates per control and treatment group, 5 µm L2). Organoids were monitored at day 0, 1, 2, 3, 4 and 7, and imaged at day 7 (DMi1 Inverted Microscope, Leica).

### 4.13 Single‐Nucleus RNA Sequencing and Cell Cross Talk Analysis

Organoids treated with 5 µm L2 for 24 h were collected and snap‐frozen in liquid nitrogen. snRNA‐seq libraries were prepared using the 10× Genomics platform, raw data were processed using the Cell Ranger toolkit (version 2.1.1) to generate the gene expression matrices, followed by downstream analysis in Seurat R package (version 4.4) [74]. Cells were filtered using the following criteria: (1) < 250 detected genes, (2) > 10% mitochondria genes, and (3) > 8000 counts. Data were normalized (NormalizeData), scaled (ScaleData), and highly variable genes (top 2000) identified (FindVariableFeatures), followed by PCA. Clustering was performed using the first 20 principal components at resolution 0.3 (FindClusters). Cell–cell communication analysis between control and treated groups was inferred using the CellChat R package [75]. Pathway‐level communication probabilities were computed with ComputeCommunProbPathway (default parameters), and network visualization was generated with netVisual_circle.

### 4.14 Immunofluorescence Staining of Ovarian Cancer Organoids

After 7 days of culture, ovarian cancer organoids were washed with PBS, embedded for cryosectioning, and sectioned. Sections were fixed in 4% paraformaldehyde for 10 min at room temperature, washed, and processed for immunostaining. Primary anti‐Ki67 antibodies (9129S, CST) were applied in PBS containing 0.1% Triton X‐100 at 4°C, followed by secondary antibody (4412S, CST) for 1 h at room temperature in the dark. Controls included assessment of tissue autofluorescence and omission of the primary antibody. Nuclei were counterstained with DAPI. Images were acquired on a Leica DMI3000B.

### 4.15 Immunofluorescence Staining of OVCAR‐3 Cells

NIH:OVCAR‐3 [OVCAR3] (SCSP‐558, Chinese Academy of Sciences Cell Bank) cells were maintained in complete medium (SCSP‐695S) and seeded in 48‐well plates. At ∼40% confluence, cells were treated with 5 µm L2. Cells were washed twice with PBS (3 min each) at room temperature for 3 min each, fixed with pre‐chilled 4% PFA for 10 min, washed twice with PBS, and stained following the same procedure as used for organoid cryosections.

### 4.16 Zebrafish Husbandry

All zebrafish procedures were approved by the Institutional Animal Care Committee of Shanghai Jiao Tong University. Zebrafish were maintained in a recirculating aquaculture system at 28°C, 80% humidity under a 14 h light/10 h dark photoperiod. Water conductivity was maintained at 500–550 µS/cm and a pH of 6.8–7.5 by supplementation with NaCl and NaHCO_3_. Wild‐type (AB strain) zebrafish embryos were collected and cultured in E3 medium at 28°C.

### 4.17 Drug Treatment in Zebrafish

Embryos were exposed to L2 in E3 medium at 10, 20, and 40 µm from 6 to 72 h post‐fertilization (hpf). For each dose, 40 healthy embryos were selected. Viability, toxicological signs, manifestations, and morphological abnormalities and deformities were evaluated every 24 h using an Olympus SZX16 stereomicroscope.

## Author Contributions

W.‐H.L. and her team (Y.‐J. Z., Y.‐X. B., D.‐Y. L., J.‐T. Y., Z.‐Y. X., Y. L., and Y.‐T. J.) conducted all plant‐related experiments, analyzed the results, and wrote the corresponding sections of the manuscript. J.W. and her team (G. G. T. and B. Z.) performed slicing and immunofluorescence staining of ovarian cancer organoids, analyzed the data of snRNA‐seq on ovary organoids, cultured ovarian cancer cell lines, and carried out compound treatments and observations. They also analyzed the experimental results and wrote the corresponding sections of the manuscript. W.‐H.L. and J.W. secured funding and drafted the manuscript. X.‐X.H. and her team (C.‐H. C.) provided the organoids, conducted compound treatments, and edited the corresponding sections of the manuscript. Y.‐X.Q. and her team (Q.‐P. Y. and W.‐H. T.) conducted experiments with rat vascular smooth muscle cells, and analyzed the results. L.‐L.J. and her team (X.‐N.W.) conducted experiments with zebrafish and analyzed the results. B.‐J. Y. provided the BY‐2 cell cultures and conducted compound treatments. Y.‐M. L. helped compare the preliminary cytotoxicity between L2 and paclitaxel. X.‐Q. J. participated in discussion and wrote parts of the manuscript.

## Funding

This research was supported by funding from the Shanghai Science and Technology Innovation Action Plan Basic Research Program 2023 (23JC1402800), the National Natural Science Foundation of China (92354301 and 32270339).

## Conflicts of Interest

The authors declare no conflicts of interest.

## Supporting information




**Supporting file 1**: advs77835‐sup‐0001‐Video S1‐S6.zip.

**Supporting file 2**: advs77835‐sup‐0002‐SuppMat.docx.

**Supporting file 3**: advs77835‐sup‐0003‐TableS1.xlsx.

**Supporting file 4**: advs77835‐sup‐0004‐Figure1‐14.pdf.

## Data Availability

The data that support the findings of this study are available on request from the corresponding author. The data are not publicly available due to privacy or ethical restrictions.
