# Supplementary material for: Plant Metabolites Screening Identifies 7‐epi‐10‐Deacetyltaxol Inhibiting Cell Division by Interfering With the Cell Cycle and Microtubule Organization
Source: Adv Sci (Weinh). 2026 Sep 16:e77835. Online ahead of print. doi: 10.1002/advs.77835 (PMC13583108; doi:10.1002/advs.77835)
Supplement: Supplementary file 1 [file ADVS-9999-e77835-s002.pdf]

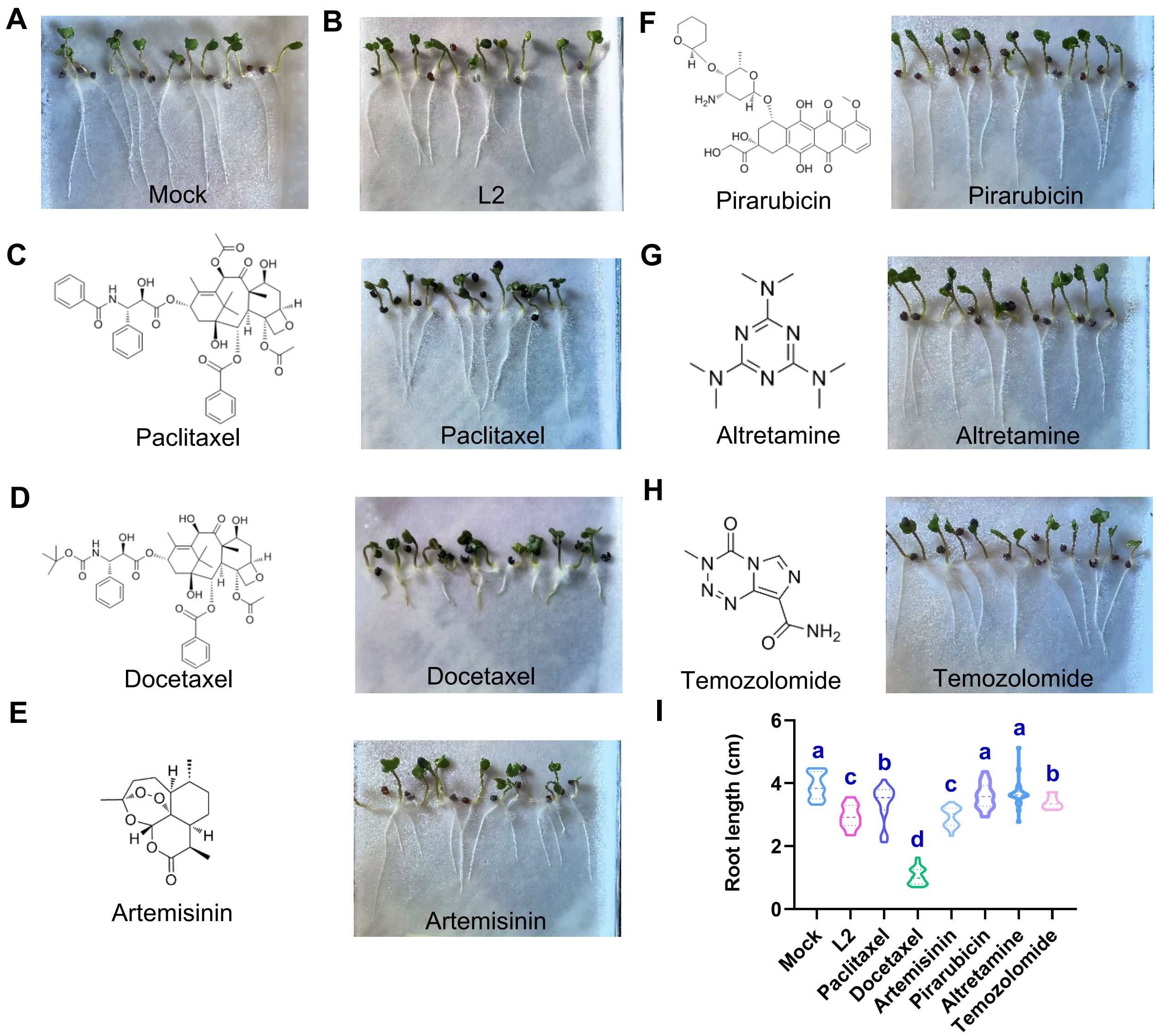

Figure S1. Effects of anticancer compounds on rapeseed seedlings. A-H. Chemical structures and representative phenotypes of rapeseed seedlings treated with 1.6 μM L2, paclitaxel, docetaxel, artemisinin, pirarubicin, altretamine and temozolomide for 3 days. Chemical structures were obtained from the web (https://www.chemsrc.com/). **I.** Quantification of primary root length, $n > 15$. Data are means ± SD. Results are representative of three independent experiments, and statistical significant was assessed by one-way ANOVA followed by Tukey's post-hoc test; different letters indicate significant differences ($p < 0.05$).

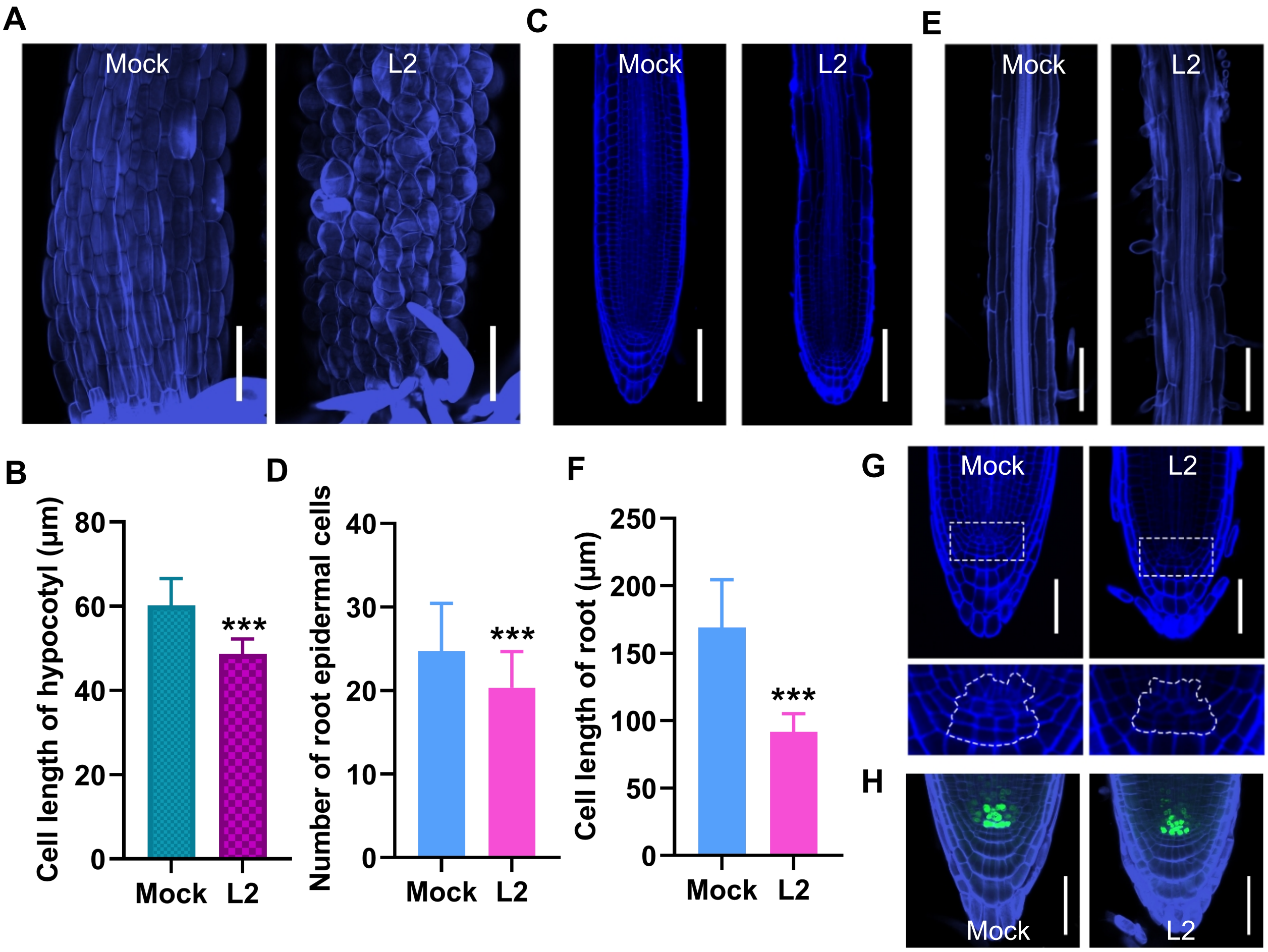

Figure S2. L2 treatment alters cell morphology in Arabidopsis seedlings. A. Epidermal cells in hypocotyls at 5 DAG. **B.** Quantification of hypocotyl cell length in **A**, n = 9. **C.** Root tips at 5 DAG. **D.** Quantification of epidermal cell number in the meristematic zone in **C**, n > 35 from 21 seedlings. **E.** Root cell profiles in the elongation zone at 5 DAG. **F.** Quantification of root epidermal cell length in **E**, n > 9. **G.** Quiescent center (QC) organization at 5 DAG. **H.** Root tips of the *pWOX5::H2B-GFP* line at 5 DAG. Seedlings were treated with mock (DMSO) or 4 µM L2 for 5 days under light in **A**, **C**, **E** , **G** and **H**. Data are means ± SD. Results are representative of three independent experiments. Statistical significance was assessed by Student's *t*-test (**B**, **D**, **F)**. *** $p < 0.001$.

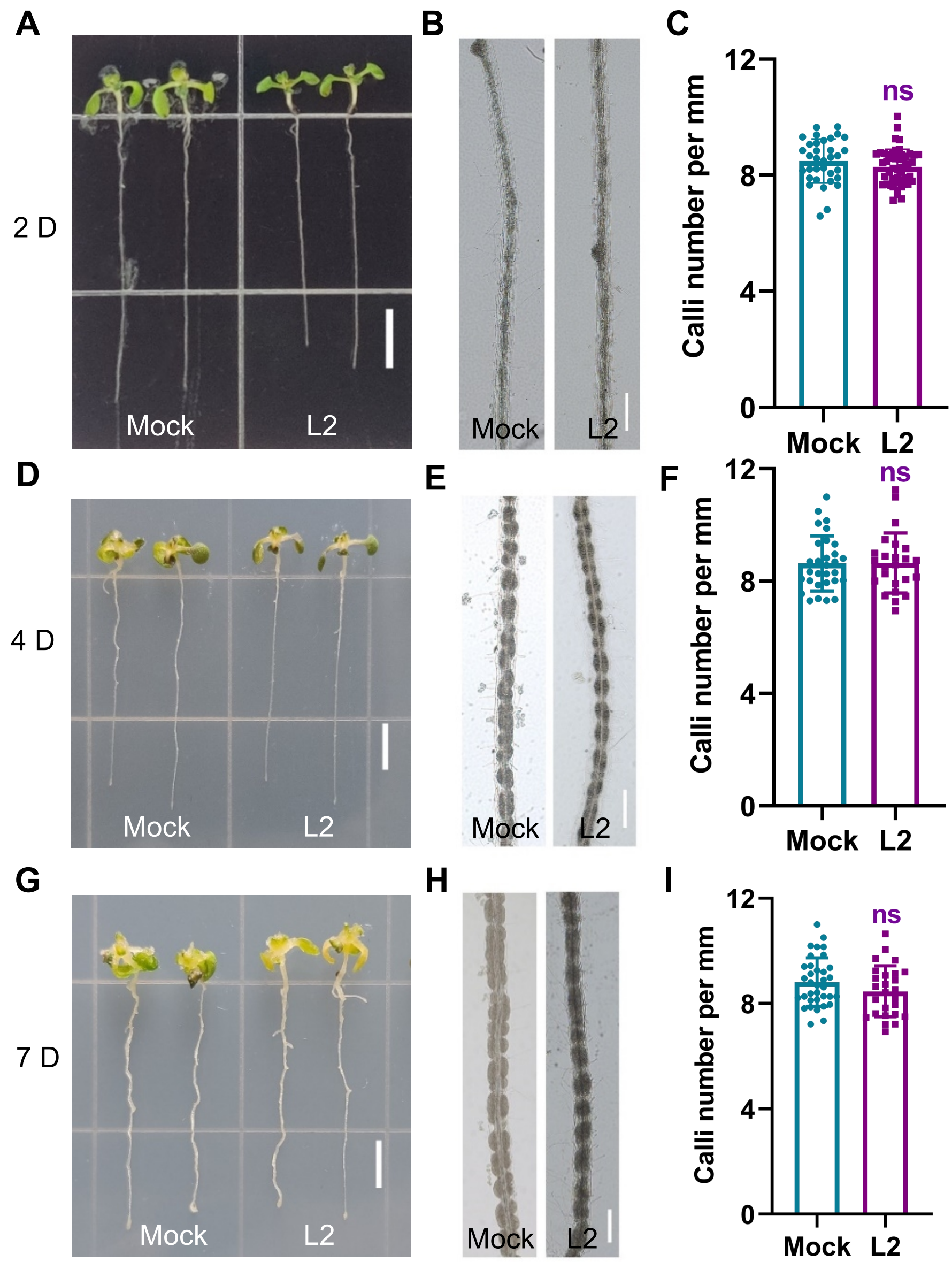

Figure S3. L2 suppresses callus initiation in Arabidopsis roots. A, **D**, **G.** Seedlings induced on callus induction medium supplemented with 4 μM L2 for 2, 4, and 7 days, respectively. **B**, **E**, **H.** Microscopic images of root callus after 2, 4, and 7-day induction. **C**, **F**, **I.** Quantification of callus number after 2, 4, and 7-day induction, n > 24. Data are means ± SD. Results are representative of three independent experiments. Statistical significance was assessed by Student's *t*-test.

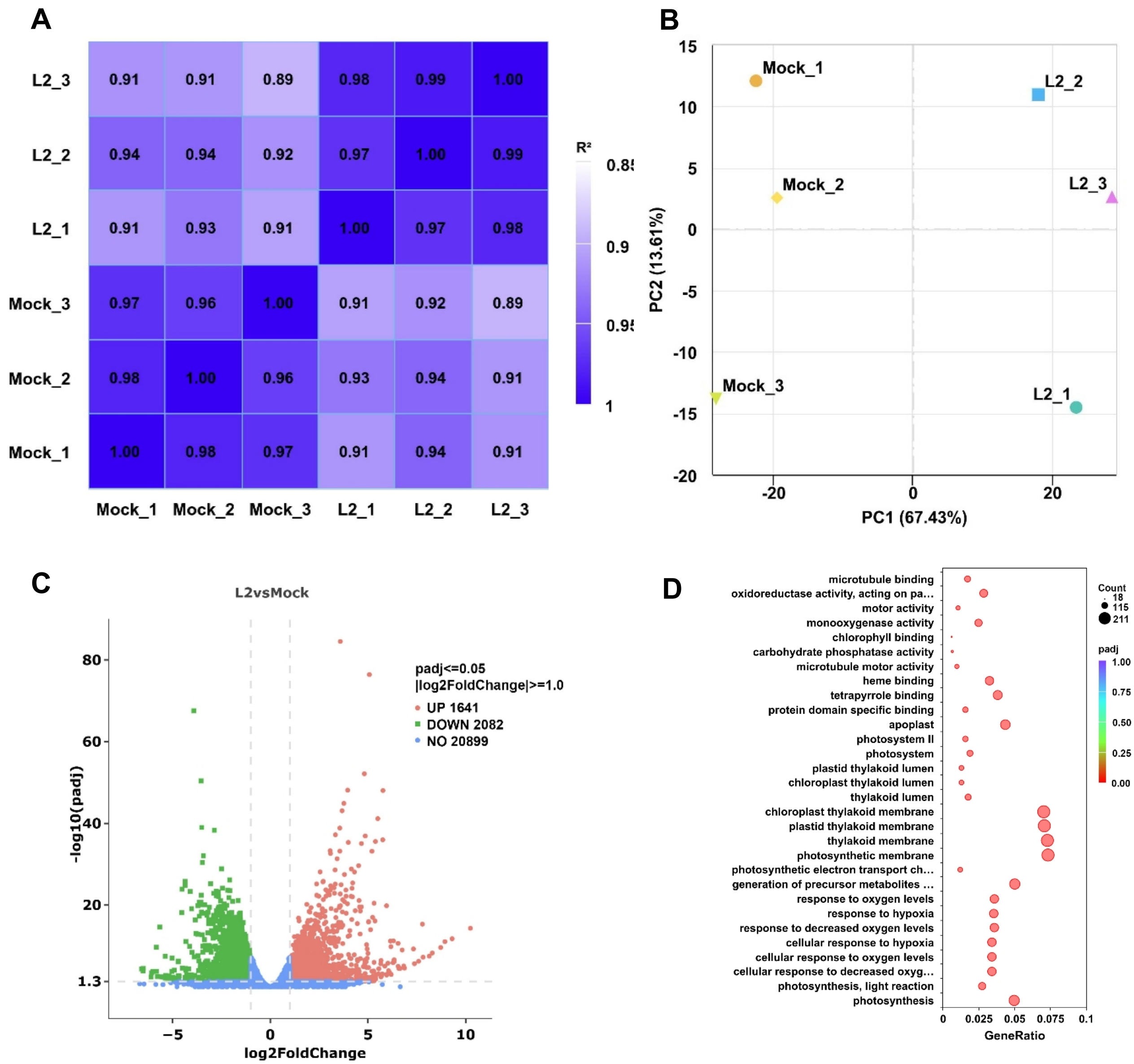

Figure S4. Transcriptomic analysis of L2-treated Arabidopsis seedlings. A. Pearson correlation matrix among RNA-seq samples from seedlings at 5 DAG. **B.** PCA of six RNA-seq samples. **C.** Volcano plot showing DEGs from RNA-seq analysis. **D.** GO enrichment analysis of DEGs (top 30 terms with the smallest p-values).

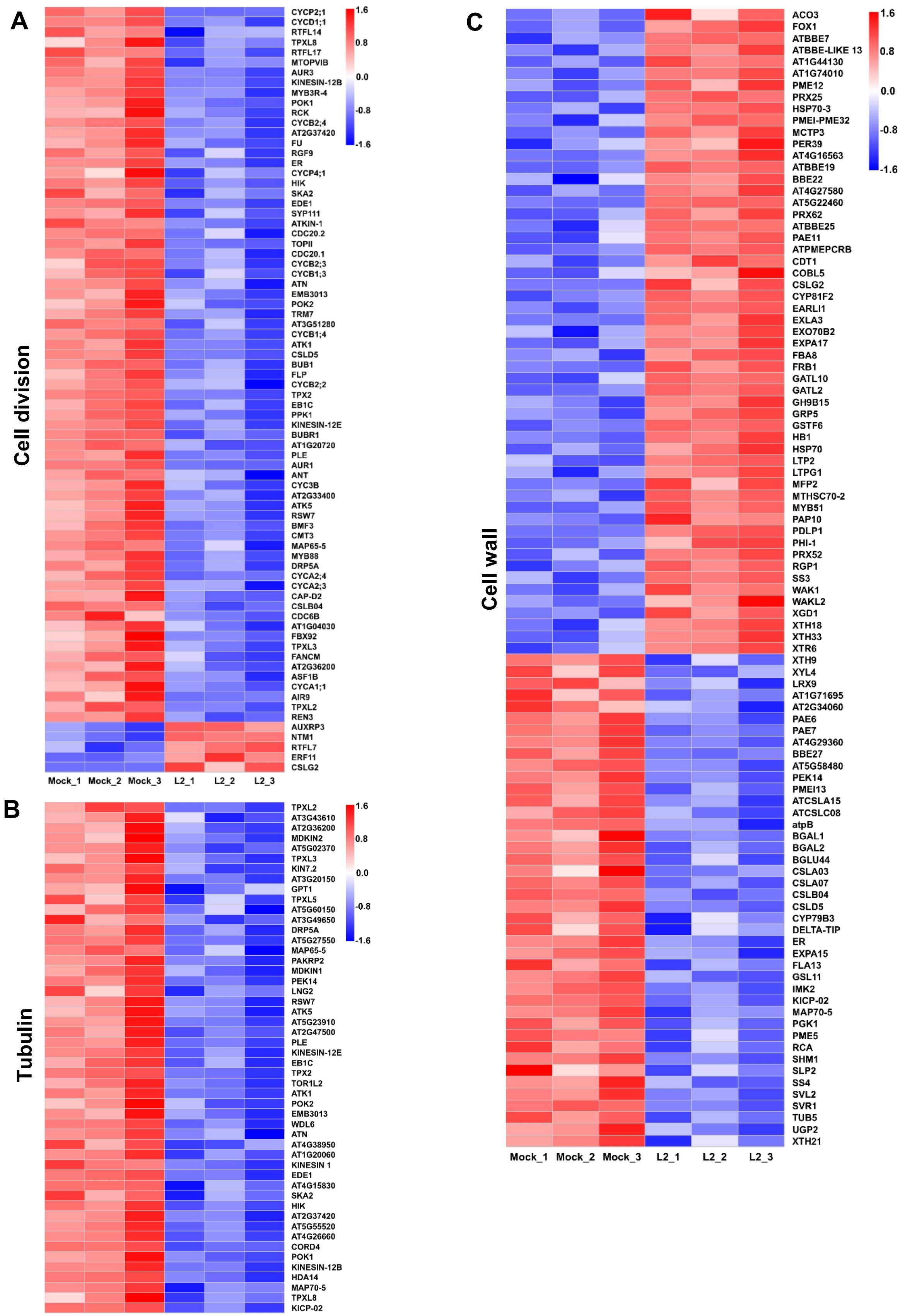

Figure S5. Heat map of RNA-seq DEGs related to cell division, tubulin, and cell wall. A-C. Hierarchical clustering heat maps of differentially expressed genes associated with cell division (76 genes), tubulins (50 genes), and cell wall (97 genes) in seedlings at 5 DAG. Red and blue indicate increased and decreased expression, respectively.

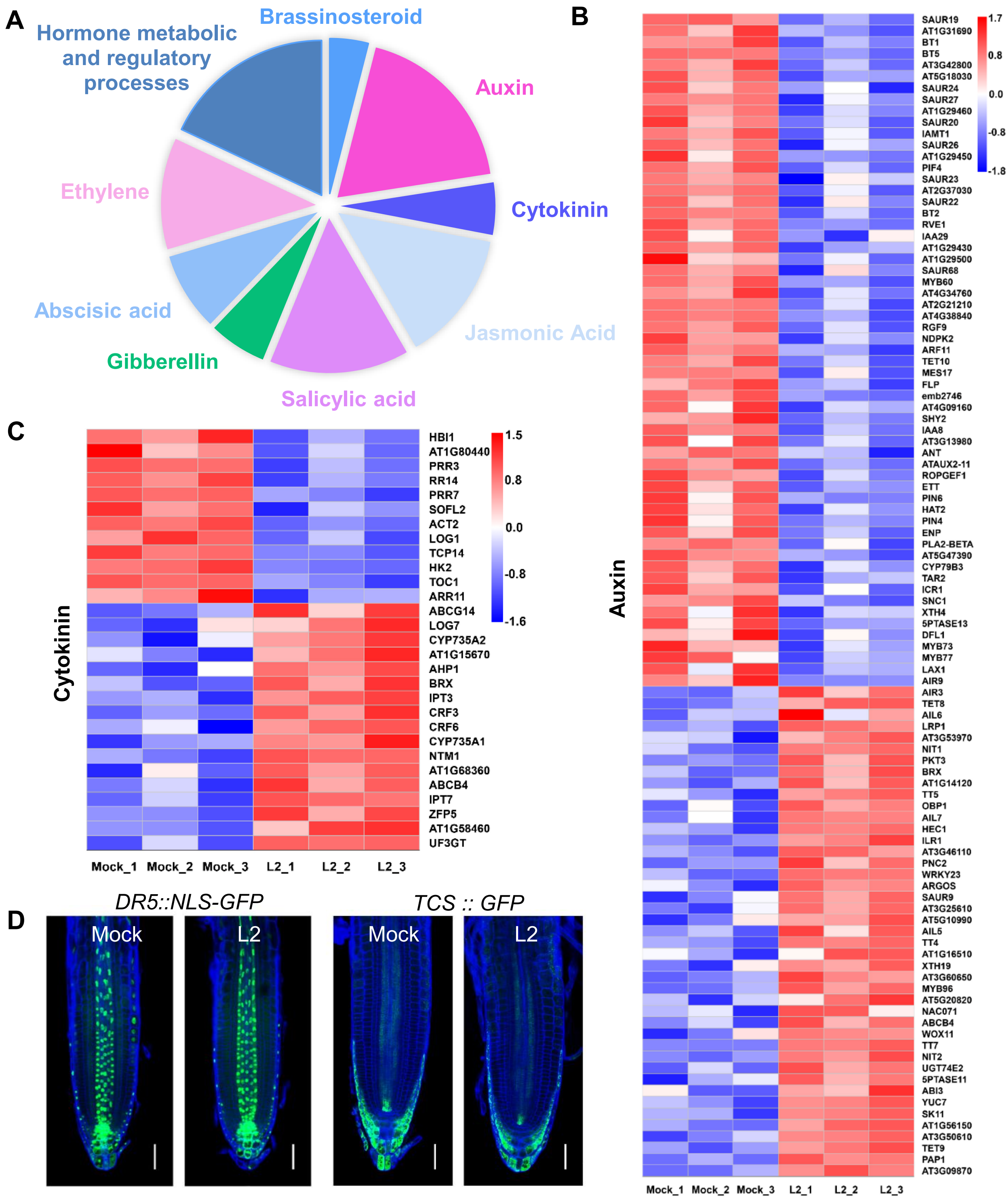

Figure S6. Hormone-related DEGs and reporter responses after L2 treatment. A. Proportion of phytohormone-related genes among DEGs from RNA-seq. **B-C**. Hierarchical clustering heat maps of differentially expressed genes associated with auxin (102 genes), and cytokinin (CK, 30 genes) in seedlings at 5 DAG. Red and blue indicate increased and decreased expression, respectively. **D.** Root fluorescence in the *DR5::NLS-GFP* and *TCS::GFP* lines at 5 DAG under mock and L2 treatment. Bar = 50 μm.

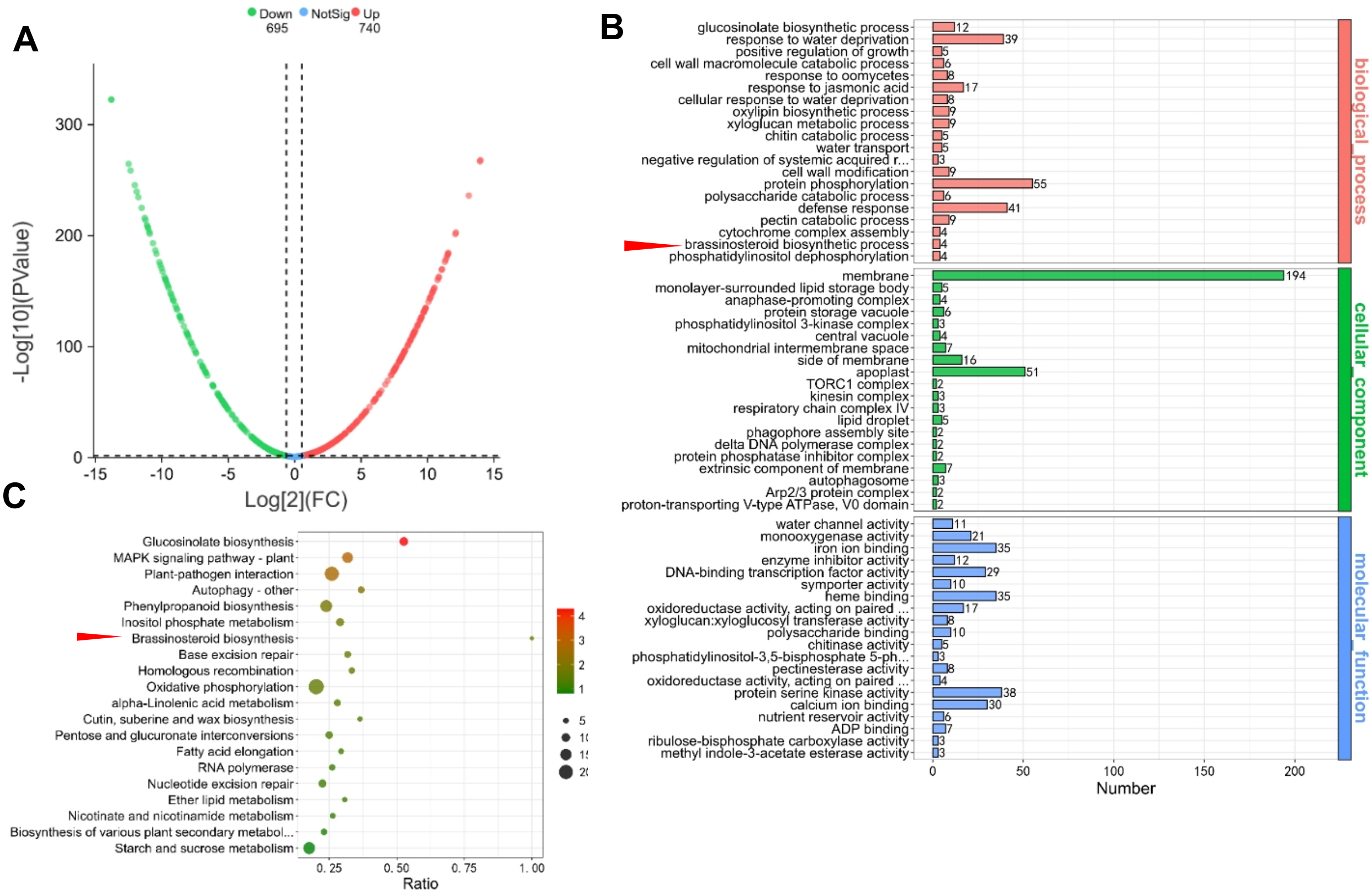

Figure S7. Differential proteomic analysis of Arabidopsis seedlings treated with L2. A. Volcano plot showing differentially expressed proteins in seedlings at 5 DAG treated with 4 μM L2. Red and blue dots indicate significantly up- and downregulated proteins, respectively (FDR < 0.05, $|log_2FC| \geq 0.58$). **B.** Gene Ontology (GO) classification of significantly altered proteins across Biological Process (BP), Molecular Function (MF), and Cellular Component (CC). **C.** KEGG pathway enrichment analysis of differentially expressed proteins. **D.** Heat map of proteins involved in BR biosynthesis and signaling pathways.

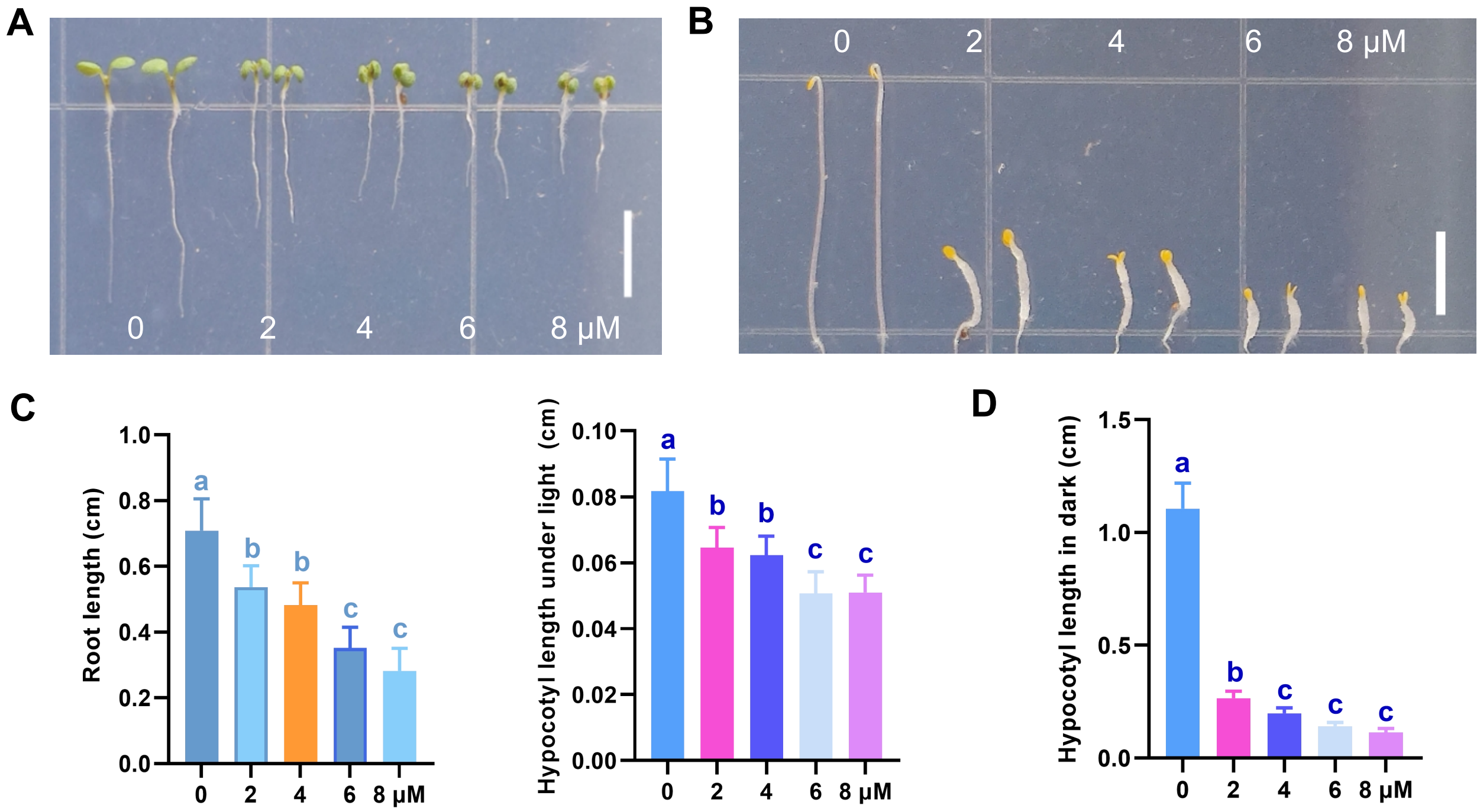

Figure S8. Paclitaxel inhibits axial growth in Arabidopsis. A-B. Representative phenotypes of seedlings at 5 DAG after paclitaxel treatment under light (**A**) and in darkness (**B**). Bar = 0.5 cm. **C.** Root and hypocotyl length under light, n > 12. **D.** Hypocotyl length in darkness, n > 9. Data are means ± SD. Results are representative of three independent experiments. Significant differences were assessed by one-way ANOVA followed by Tukey's post-hoc test; different letters indicate significant differences ($p < 0.05$).

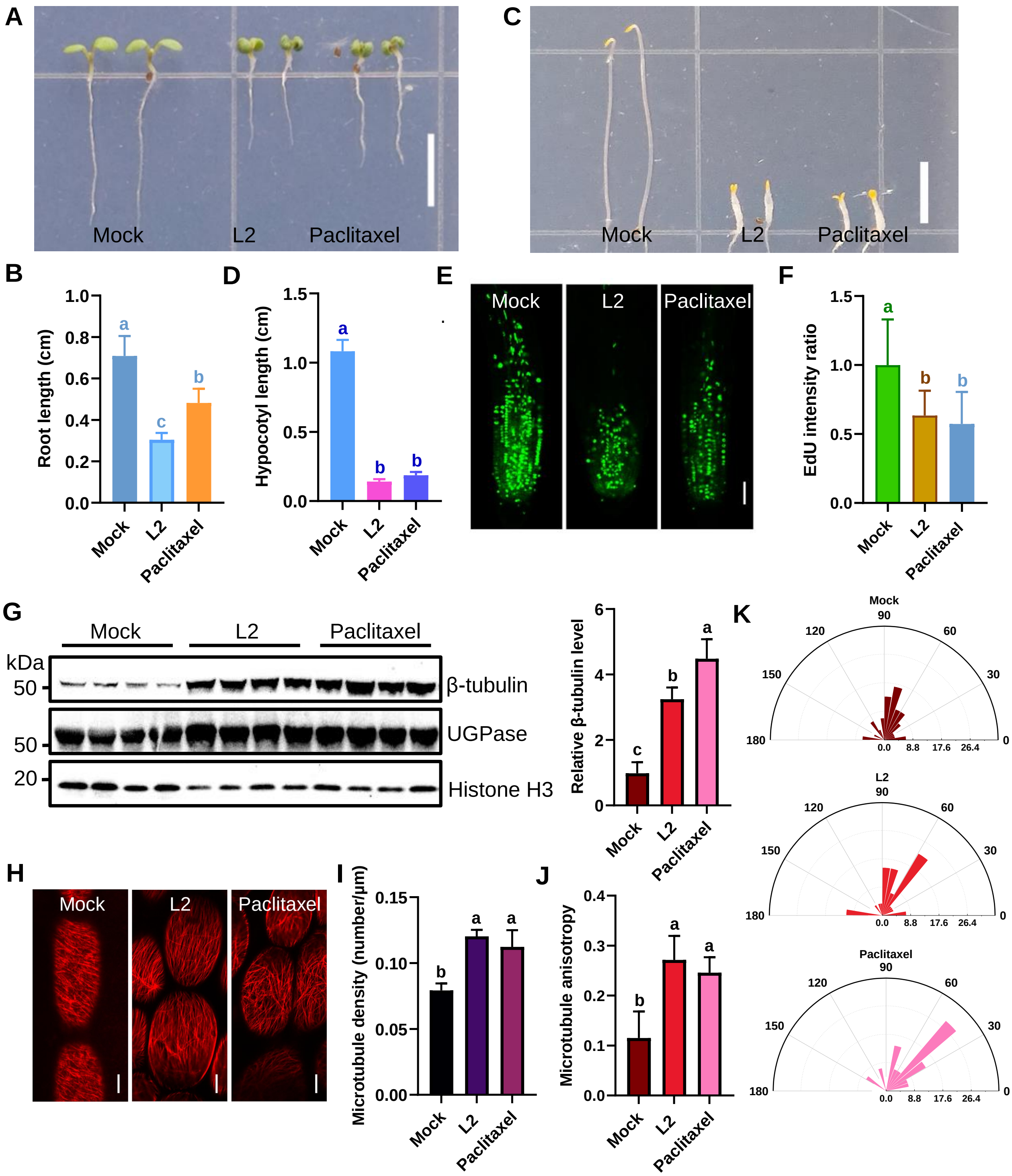
A
Mock
L2
Paclitaxel
C
Mock
L2
Paclitaxel
B
Root length (cm)
1.0
0.8
0.6
0.4
0.2
0.0
a
c
b
Mock
L2
Paclitaxel
D
Hypocotyl length (cm)
1.5
1.0
0.5
0.0
a
b
b
Mock
L2
Paclitaxel
E
Mock
L2
Paclitaxel
F
EdU intensity ratio
1.5
1.0
0.5
0.0
a
b
b
Mock
L2
Paclitaxel
G
Mock
L2
Paclitaxel
kDa
50
50
20
β-tubulin
UGPase
Histone H3
Relative β-tubulin level
6
4
2
0
c
b
a
Mock
L2
Paclitaxel
K
Mock
L2
Paclitaxel
90
120
60
150
30
180
0
0.0
8.8
17.6
26.4
H
Mock
L2
Paclitaxel
I
Microtubule density (number/μm)
0.15
0.10
0.05
0.00
b
a
a
Mock
L2
Paclitaxel
J
Microtubule anisotropy
0.4
0.3
0.2
0.1
0.0
b
a
a
Mock
L2
Paclitaxel

Figure S9. Comparison of inhibitory effects of paclitaxel and L2 in Arabidopsis. A. Representative phenotypes of seedlings at 5 DAG under light after L2 or paclitaxel treatment. Bar = 0.5 cm. **B.** Root length under light in **A**, $n > 15$. **C.** Representative phenotypes of seedlings at 5 DAG in darkness after L2 or paclitaxel treatment. Bar = 0.5 cm. **D.** Hypocotyl length in darkness in **C**, $n > 15$. Wild type seedlings were treated with 4 μM L2 or paclitaxel under light or in darkness. **E.** EdU staining of proliferating cells in roots of wild type seedlings at 3 DAG. Bar = 50 μm. **F.** Quantification of EdU incorporation shown as the intensity ratio measured in ImageJ, $n > 15$. **G.** Western blotting of β-tubulin in total extracts; UGPase and histone H3 were used as cytoplasmic and nuclear loading controls, respectively. 4 independent biological replicates are shown for each treatment. Quantification of β-tubulin protein levels from all 4 biological replicates is shown to the right, normalized to UGPase and relative to mock. Data are mean ± SD; statistical significance was determined by one-way ANOVA followed by Tukey's post-hoc test, and different letters indicate significant differences ($p < 0.05$). **H.** Representative cortical microtubule arrays in hypocotyl epidermal cells of the *TUB6-mCherry* line at 5 DAG under light after L2 or paclitaxel treatment. Bar = 10 μm. **I–K.** Quantification of microtubule density (**I**), anisotropy (**J**), and orientation (**K**) from **H**. For (**I**) and (**J**), data are mean ± SD from 3 biological replicates per treatment (≥10 cells per replicate); statistical tests were performed on replicate-level means. Significant differences were assessed by one-way ANOVA followed by Tukey's post-hoc test; different letters indicate significant differences ($p < 0.05$). For (**K**) rose diagrams show the frequency distribution of microtubule orientation angles relative to the cell long axis, measured using FibrilTool and binned into 10° intervals (0–180°); >30 cells per treatment from 3 biological replicates are shown for visualization only.

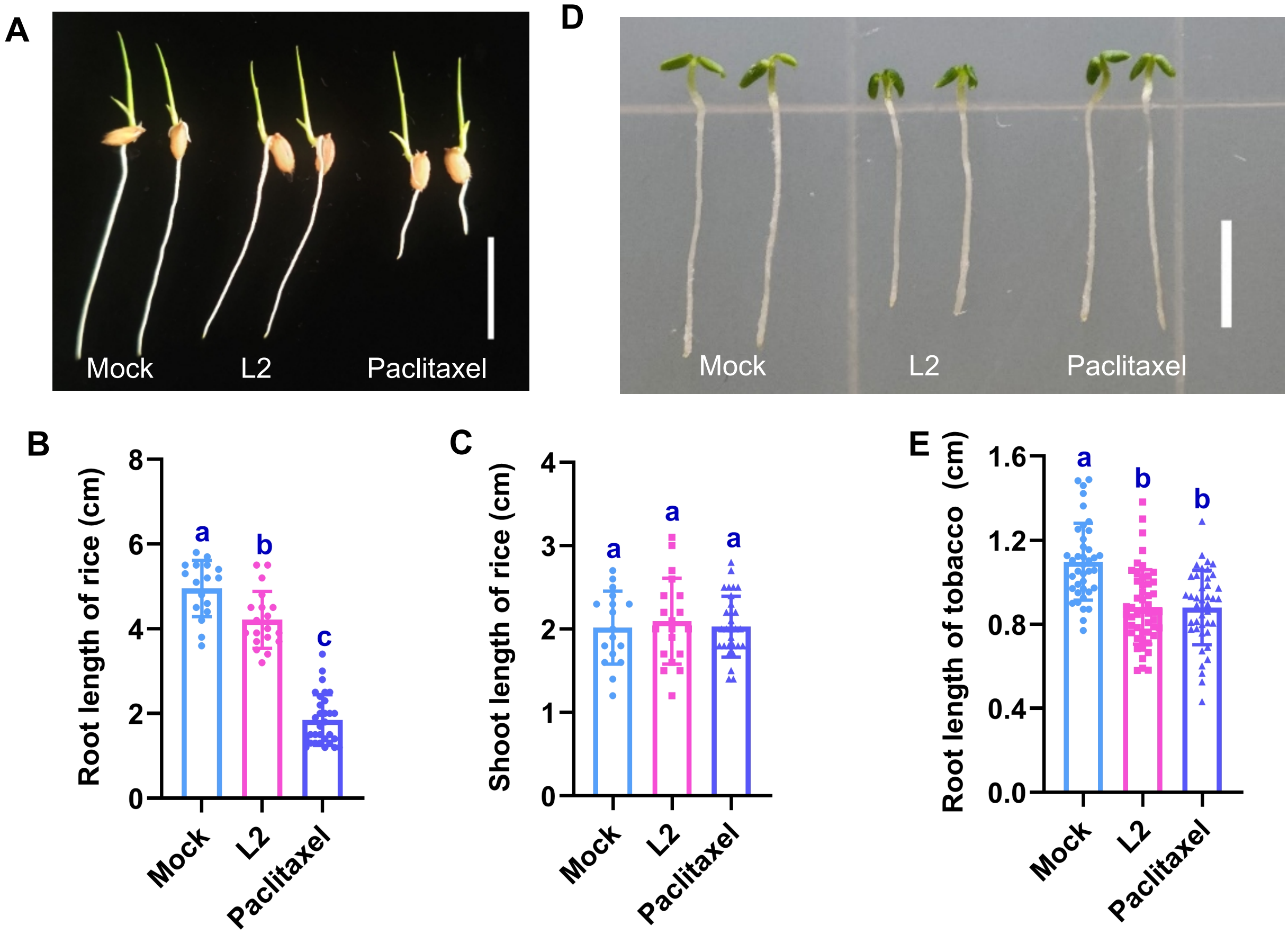

Figure S10. Comparison of inhibitory effects of L2 and paclitaxel in rice and tobacco. A. Representative phenotypes of 8-day-old rice seedlings grown under light. Wild type ZH11 seedlings at 4 DAG were treated with 4 μM L2 or paclitaxel for 4 days. Bar = 2 cm. **B-C.** Root and shoot lengths of rice seedlings, n > 15. **D.** Representative phenotype of 11-day-old tobacco seedlings grown under light. Seedlings at 2 DAG were treated with 4 μM L2 or paclitaxel for 9 days. **E.** Root length of tobacco seedlings, n > 39. Data are means ± SD. Results are representative of three independent experiments, significant differences were assessed by one-way ANOVA followed by Tukey's post-hoc test, different letters indicate significant differences ($p < 0.05$).

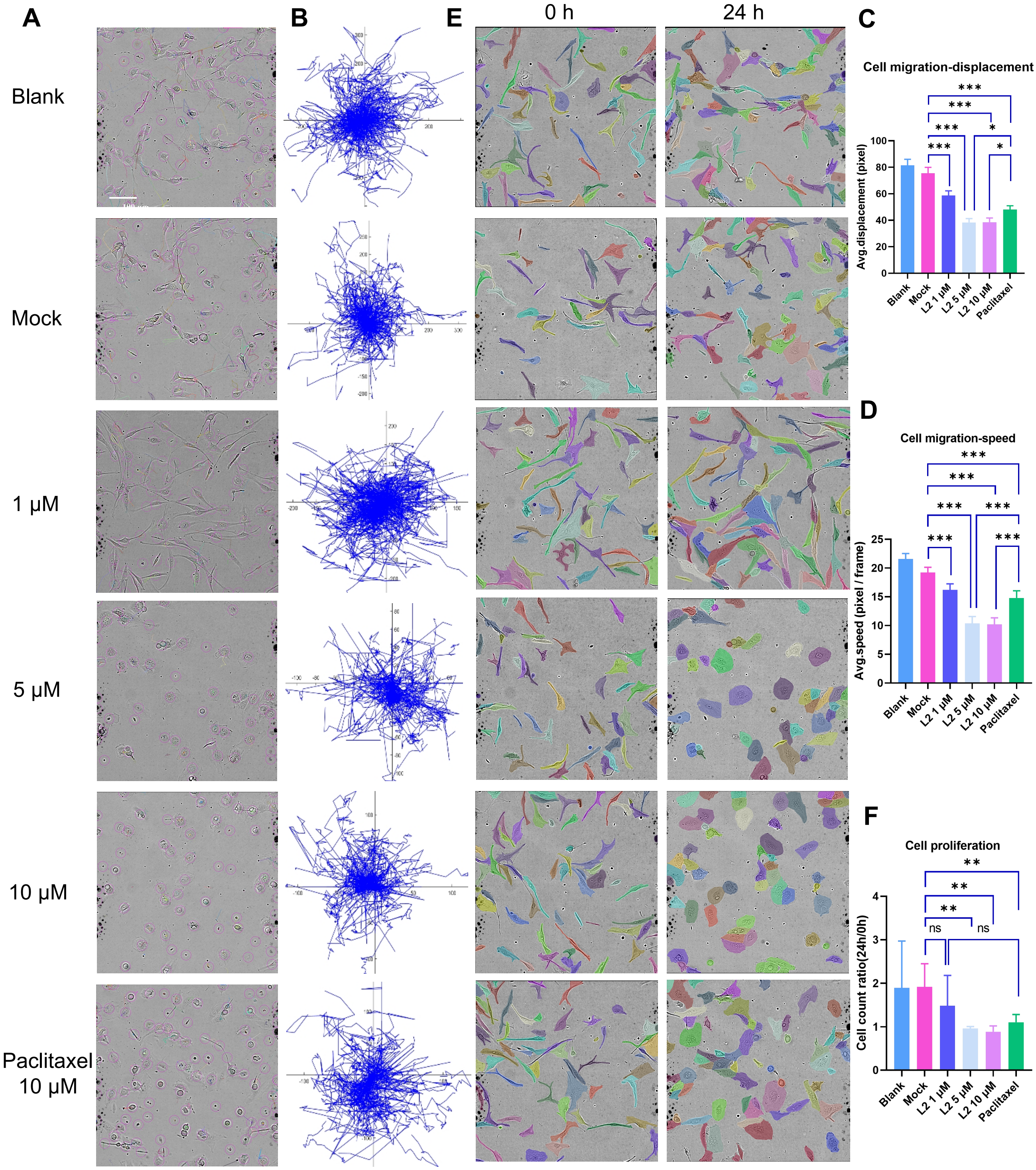

Figure S11. L2 inhibits proliferation and motility of rat vascular smooth muscle cells. A. Representative cell outlines at 24 h. Cells were treated with no drug (Blank), mock (DMSO), and L2 (1, 5, and 10 μM), or paclitaxel (10 μM). **B.** Cell migration trajectories overlaid as blue tracks. **C, D.** Quantification of migration displacement (**C**) and migration speed (**D**). **E.** Colored polygons showing cell masks at 0 and 24 h. **F.** Quantification of cell proliferation. Data are means ± SE, n = 3 biological replicates, statistical significance was assessed by one-way ANOVA followed by Tukey's post-hoc test (**C**, **D**, **F**).

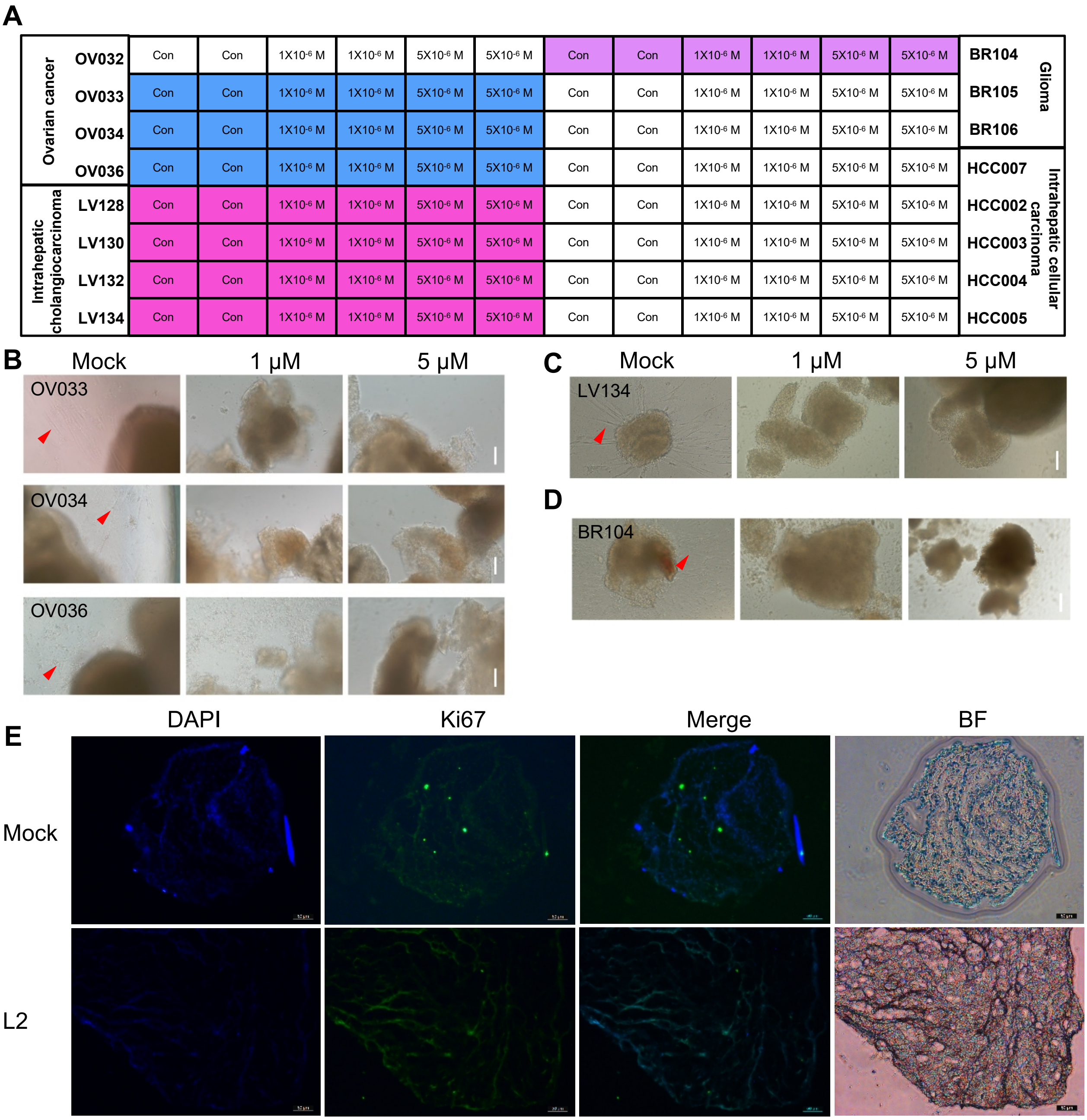

Figure S12. L2 inhibits growth of the organoids derived from multiple human cancers. A. Schematic diagram of organoids treated with different concentrations of L2, responding individuals are highlighted. **B.** Representative ovarian cancer organoids after 5-day L2 treatment. Bar = 100 μm. **C.** Representative intrahepatic cholangiocarcinoma organoid after 3-day L2 treatment. Bar = 100 μm. **D.** Representative glioma organoid after 3-day L2 treatment. Bar = 100 μm. Red arrowheads indicate newly detached/free cells at organoid edges in **B-D**. **E.** Ki67 immunostaining with DAPI nuclear staining in cryosections of ovarian cancer organoids. Bar = 50 μm.

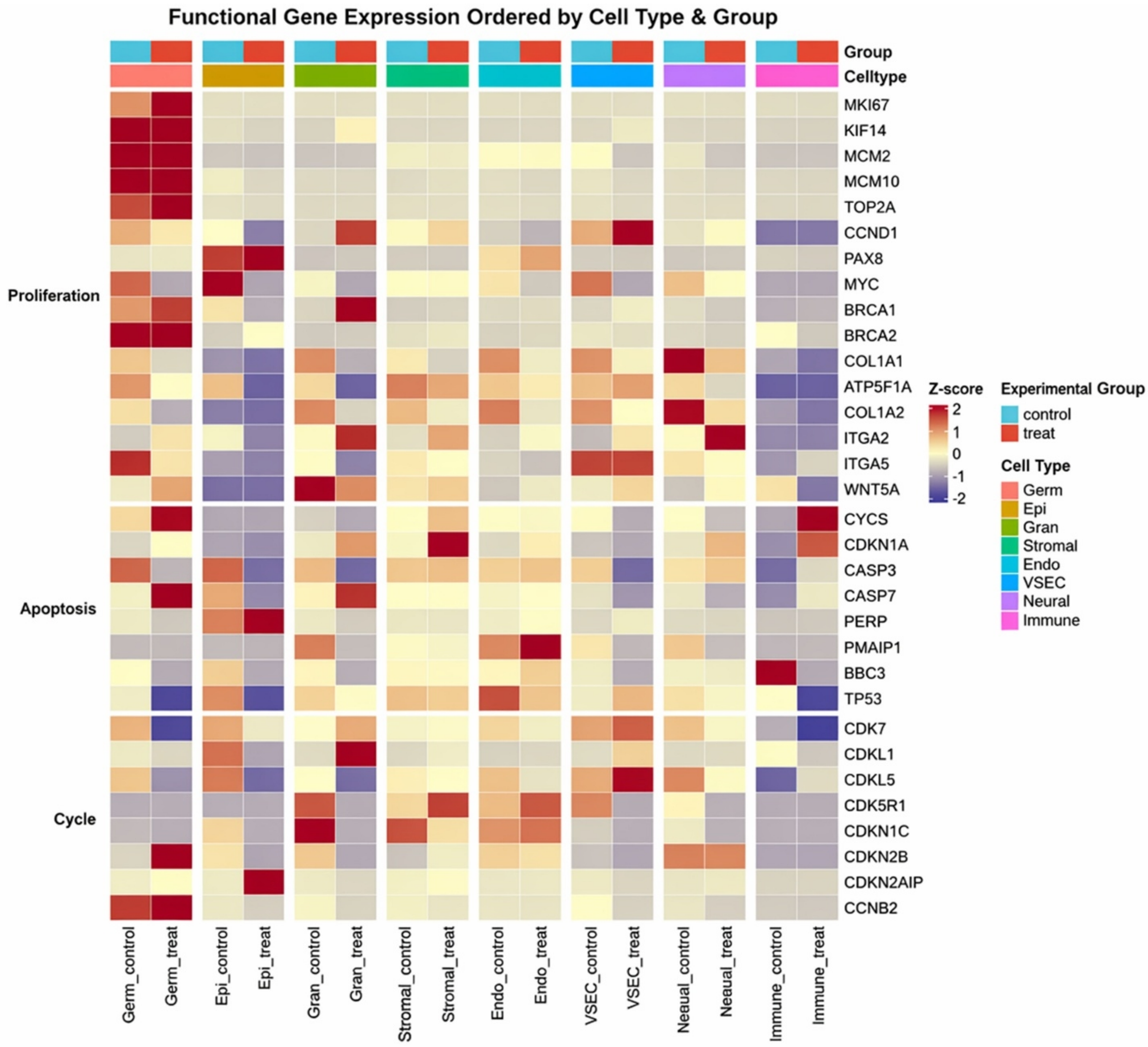

Figure S13. Cell type-resolved expression of proliferation-, apoptosis-, and cell cycle-related genes in OVA-64 organoids following L2 treatment. Heatmap showing Z-score-normalized expression of curated marker genes representing three functional gene sets-proliferation/viability, apoptosis and cell-cycle regulation-across the eight major cell types identified by snRNA-seq. For each cell type, mean expression is shown separately for the mock control and L2-treated groups. Rows are grouped by functional category and columns are grouped by cell type and treatment condition. Color scale indicates row-wise Z-score, with red representing relatively higher expression and blue representing relatively lower expression within each gene.

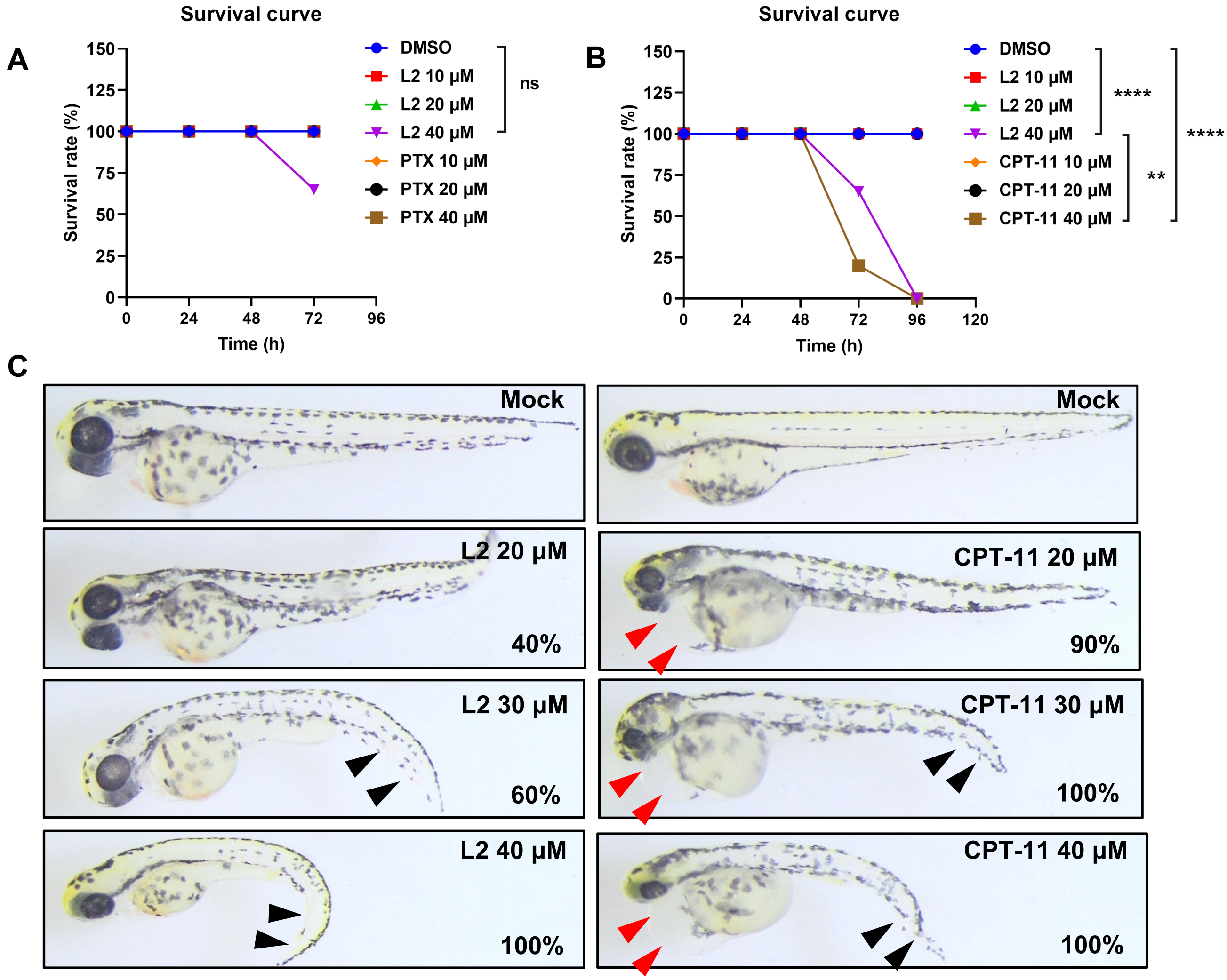

Figure S14. Toxicity assessment of L2 in zebrafish embryos. A. Survival of embryos at 72 h post-fertilization (hpf) after exposure to L2 or paclitaxel at 10, 20, and 40 µM. **B.** Survival of embryos at 96 hpf after exposure to L2 or irinotecan (CPT-11) at 10, 20, and 40 µM. **C.** Representative phenotypic abnormalities at 72 hpf. Drug-exposed embryos exhibit curved tails (black arrows) and pericardial edema (red arrows) compared with mock (DMSO). Statistical significance for survival comparisons was assessed by the log-rank (Mantel-Cox) test (**A**, **B**). ns, not significant; **, $p < 0.01$; ****, $p < 0.0001$.
