# Supplementary material for: Plant Metabolites Screening Identifies 7‐epi‐10‐Deacetyltaxol Inhibiting Cell Division by Interfering With the Cell Cycle and Microtubule Organization
Source: Adv Sci (Weinh). 2026 Sep 16:e77835. Online ahead of print. doi: 10.1002/advs.77835 (PMC13583108; doi:10.1002/advs.77835)
Supplement: Supplementary file 2 [file ADVS-9999-e77835-s003.docx]

**Figure 1. L2 inhibits axial growth in Arabidopsis. A.** Workflow of chemical screening using a plant-derived compound library in rapeseed Zhongshuang 11. **B**. Chemical structures of compounds in group 3 as an example for the grouping principle. **C**. Results of grouped screening. The height of each column represents the relative root length of the respective group compared with the mock group treated with DMSO. All colored columns indicate compound groups that effectively inhibited root growth, as judged by visual inspection, and red columns indicate groups reduced by at least 25% through actual measurement. (n = 12) **D**. Results of treatments with every single compound from the effective groups (red columns in **C**). Colored points represent the relative length and blurred points indicate the measured values. Reported drugs or phytohormones are highlighted in blue, and the compound 7-epi-10-deacetyltaxol for further investigation is highlighted in red. **E.** Representative phenotypes of wild-type seedlings at 5 DAG grown under light and treated with L2 (0, 2, 4, 6, and 8 µM). Bar = 0.5 cm. **F.** Quantification of root length in **E**, n > 15. **G.** Representative phenotypes of wild-type seedlings at 5 DAG grown in darkness and treated with L2 (0, 2, 4, 6, and 8 µM). Bar = 0.5cm. **H.** Quantification of hypocotyl length in **G**, n > 15. **I.** Petiole phenotype of rosette leaves. Bar = 2 cm. **J.** Quantification of petiole length in **I**, n > 30. **K.** Inflorescence axis phenotype after continuous application of L2 to the SAM for 2 weeks. **L.** Quantification of inflorescence axis length in **K**, n > 15. **M.** Pistil and placenta phenotypes after L2 treatment for 24 h. **N.** Quantification of ovule number per placenta in **M**, n > 15. Data are means ± SD. Results are representative of three independent experiments. Statistical significance was assessed by Student’s *t*-test (**J**, **L**, **N**), ** p < 0.01, *** p < 0.001; or by one-way ANOVA followed by Tukey’s post-hoc test (**F**, **H**), where different letters indicate significant differences (*p* < 0.05).

**Figure 2. L2 suppresses cell division in Arabidopsis roots. A.** EdU staining of proliferating cells in root tips at 3 DAG. Bar = 50 μm. **B.** Quantification of EdU incorporation (intensity ratio). **C, E.** Representative fluorescence images of root tips at 5 DAG expressing the cytokinesis marker *pKNOLLE::GFP-KNOLLE* (**C**) and the G1/S marker *HIS4::D box-Venus* (**E**) lines. Cell outlines were counterstained with Fluorescent Brightener 28 (FB28). Bar = 50 μm. **D, F.** Quantification of *pKNOLLE::GFP-KNOLLE* (**D**) and *HIS4::D box-Venus* (**F**) fluorescence intensity. **G.** Relative transcript levels of cell-division/cell-cycle–associated genes including *CDKA;1*, *CYCD3;1*, *E2FA*, *PCNA1*, *MCM3*, *CDKB1;1*, *MYB3R4*, *KNOLLE*, *HIS4* and *AUR1* in roots at 5 DAG. Data are presented as means ± SD for (**B**, **D**, **F**) or means ± SE for (**G**) from three independent biological replicates (n = 3). For imaging quantification in **B**, **D**, **F**, at least 5, 6, and 8 roots were analyzed per treatment in each biological replicate, respectively. Statistical significance between treatments was assessed by two-tailed Student’s *t*-test; * *p* < 0.05, ** *p* < 0.01.

**Figure 3. L2 inhibits proliferation of PSB-D suspension cells and BY-2 cultures. A.** Optical density of PSB-D(MM2d) cultures after 2-day treatment. **B**. Fresh weight of PSB-D(MM2d) cultures after 7-day treatment. **C.** EdU staining of PSB-D cells after 1-day treatment. Cell outlines were stained with FB28. Bar = 100 μm. **D.** Quantification of EdU incorporation (intensity ratio). **E.** Evans blue staining of PSB-D suspension cells after 4 μM L2 treatment. Bar = 100 μm. **F.** Neutral red staining of PSB-D cells. Bar = 100 μm. **G.** Representative growth phenotypes of BY-2 cultures on solid medium after 2- and 17-day treatments. **H.** Increase in fresh weight of BY-2 cultures after 21-day treatment. **I.** Representative BY-2 cell morphology with FB28-stained cell outlines after L2 treatment, four cells are traced for visualization. Bar = 100 μm. Unless otherwise indicated, PSB-D cultures were treated with 4 µM L2. Data are presented as means ± SE. The sample size (n) represents the number of independent biological replicates (n = 5 for **A** and **B**; n = 3 for **D** and **H**). For image quantification in **D**, at least 7 cultures were analyzed per treatment in each biological replicate. Statistical significance was assessed by two-tailed Student’s *t*-test, * *p* < 0.05, ** *p* < 0.01.

**Figure 4. L2 alters BR responses in Arabidopsis. A.** Heat map of 19 BR biosynthesis, metabolism, and signaling-related DEGs. Red and blue indicate up- and downregulation, respectively. **B.** Primary root length of *Col* and *det2* seedlings at 12 DAG treated with L2 (0, 4, 6, and 8 μM), n > 24. **C-D.** Relative root length (L2/Mock ratio) of *Col* vs. *DWF4-OX* (**C**) and *Col* vs. *bri1-11*6 (**D**) in seedlings at 7 DAG treated with 4 μM L2, n = 3 biological replicates. **E.** Relative transcript levels of *CPD* and *DWF4* in wild type seedlings at 5 DAG treated with 10 μM L2 for 0, 0.5, 1, 3, and 4 h, n = 3 biological replicates. **F, H.** Representative BZR1-YFP (**F**) and BES1-GFP (**H**) fluorescence in roots at 5 DAG. Cell outlines were counterstained with FB28. Bar = 50 μm. **G.** Quantification of BZR1-YFP fluorescence intensity per cell in epidermal cells of the meristematic zone, n > 74 cells from 8 seedlings. **I.** Quantification of BES1-GFP fluorescence intensity per cell in epidermal cells of the meristematic zone, n > 56 cells from 6 seedlings. **J.** Heat map of 10 proteins related to brassinosteroid biosynthesis, metabolism, and signaling pathways identified by proteomic analysis. Data in (**B, C, D, G,** **I**) are means ± SD or means ± SE (**E**), from three independent biological replicates. Statistical significance was assessed by two-way ANOVA followed by Tukey's multiple comparison test (**B**), two-tailed ratio paired *t*-test (**C, D**), or Student’s *t*-test (**E**, **G**, **I**); different letters indicate significant differences (p < 0.05), * p < 0.05, ** *p* < 0.01, *** *p* < 0.001.

**Figure 5. L2 disrupts cortical microtubule organization. A-B.** Representative cortical microtubule arrays in hypocotyl (**A**) and root (**B**) epidermal cells of the *TUB6-mCherry* line after 6 h treatments as indicated (mock, 10 μM L2, 10 nM eBL, 10 μM L2 + 10 nM eBL, 2 μM BRZ, and 10 μM L2 + 2 μM BRZ respectively). Bar = 10 μm. **C.** Mitotic spindle organization in the largest optical section of the root meristem after 6 h treatment with mock, 10 μM L2, 10 nM eBL, and 10 nM eBL+10 μM L2, respectively. Bar = 25 μm. **D-F.** Quantification of microtubule density (**D**), anisotropy (**E**), and orientation (**F**) from **A**. For **D** and **E**, data are mean ± SD from 3 biological replicates per treatment (≥10 cells per replicate); statistical tests were performed on replicate-level means. Rose diagrams (**F**) show the frequency distribution of microtubule orientation angles relative to the cell long axis, measured using FibrilTool and binned into 10° intervals (0–180°); >30 cells per treatment from 3 biological replicates are shown for visualization only. **G.** Western blotting of BIN2 and CYCD3;1 in total cell extracts under the indicated treatments (DMSO, DMSO + eBL, L2, and L2 + eBL). UGPase was used as a cytoplasmic loading control. **H.** Quantification of BIN2 and CYCD3;1 protein levels in (**G**), normalized to UGPase and shown relative to mock. Data are mean ± SD from 3 independent biological replicates. For **D**, **E**, and **H**, statistical significance was determined by one-way ANOVA with Tukey’s post hoc test; different letters indicate significant differences (P < 0.05).

**Figure 6. L2 inhibits growth of ovarian cancer organoids and cell line. A.** Representative ovarian cancer organoids after 7-day treatment with 5 μM L2. Two organoids showing pronounced growth inhibition are boxed. Red arrowheads indicate detached/single cells at organoid edges. The bars are shown below each image column. **B.** UMAP visualization of snRNA-seq data, colored by eight major cell types, each cell cluster includes a pie chart indicating the proportion of control versus L2-treated cells. **C.** Circos plot depicting inferred intercellular communication among clusters in control and L2-treated groups. **D.** Ki67 immunostaining with DAPI nuclear counterstaining in an ovarian cancer cell line after 24 h of treatment with L2. Bar = 100 μm.

**Figure S1. Effects of anticancer compounds on rapeseed seedlings. A-H.** Chemical structures and representative phenotypes of rapeseed seedlings treated with 1.6 μM L2, paclitaxel, docetaxel, artemisinin, pirarubicin, altretamine and temozolomide for 3 days. Chemical structures were obtained from the web (<https://www.chemsrc.com/>). **I.** Quantification of primary root length, n > 15. Data are means ± SD. Results are representative of three independent experiments, and statistical significant was assessed by one-way ANOVA followed by Tukey’s post-hoc test; different letters indicate significant differences (*p* < 0.05).

**Figure S2. L2 treatment alters cell morphology in Arabidopsis seedlings. A.** Epidermal cells in hypocotyls at 5 DAG. **B.** Quantification of hypocotyl cell length in **A**, n = 9. **C.** Root tips at 5 DAG. **D.** Quantification of epidermal cell number in the meristematic zone in **C**, n > 35 from 21 seedlings. **E.** Root cell profiles in the elongation zone at 5 DAG. **F.** Quantification of root epidermal cell length in **E**, n > 9. **G.** Quiescent center (QC) organization at 5 DAG. **H.** Root tips of the *pWOX5::H2B-GFP* line at 5 DAG. Seedlings were treated with mock (DMSO) or 4 μM L2 for 5 days under light in **A**, **C**, **E**, **G** and **H**. Data are means ± SD. Results are representative of three independent experiments. Statistical significance was assessed by Student’s *t*-test (**B**, **D**, **F)**. *** *p* < 0.001.

**Figure S3. L2 suppresses callus initiation in Arabidopsis roots. A, D, G.** Seedlings induced on callus induction medium supplemented with 4 μM L2 for 2, 4, and 7 days, respectively. **B, E, H.** Microscopic images of root callus after 2, 4, and 7-day induction. **C, F, I.** Quantification of callus number after 2, 4, and 7-day induction, n > 24. Data are means ± SD. Results are representative of three independent experiments. Statistical significance was assessed by Student’s *t*-test.

**Figure S4. Transcriptomic analysis of L2-treated Arabidopsis seedlings. A.** Pearson correlation matrix among RNA-seq samples from seedlings at 5 DAG. **B.** PCA of six RNA-seq samples. **C.** Volcano plot showing DEGs from RNA-seq analysis. **D.** GO enrichment analysis of DEGs (top 30 terms with the smallest p-values).

**Figure S5. Heat map of RNA-seq DEGs related to cell division, tubulin, and cell wall. A-C.** Hierarchical clustering heat maps of differentially expressed genes associated with cell division (76 genes), tubulins (50 genes), and cell wall (97 genes) in seedlings at 5 DAG. Red and blue indicate increased and decreased expression, respectively.

**Figure S6. Hormone-related DEGs and reporter responses after L2 treatment. A.** Proportion of phytohormone-related genes among DEGs from RNA-seq. **B-C.** Hierarchical clustering heat maps of differentially expressed genes associated with auxin (102 genes), and cytokinin (CK, 30 genes) in seedlings at 5 DAG. Red and blue indicate increased and decreased expression, respectively. **D.** Root fluorescence in the *DR5::NLS-GFP* and *TCS::GFP* lines at 5 DAG under mock and L2 treatment. Bar = 50 μm.

**Figure S7. Differential proteomic analysis of Arabidopsis seedlings treated with L2. A.** Volcano plot showing differentially expressed proteins in seedlings at 5 DAG treated with 4 µM L2. Red and blue dots indicate significantly up- and downregulated proteins, respectively (FDR < 0.05, |log₂FC| ≥ 0.58). **B.** Gene Ontology (GO) classification of significantly altered proteins across Biological Process (BP), Molecular Function (MF), and Cellular Component (CC). **C.** KEGG pathway enrichment analysis of differentially expressed proteins. **D.** Heat map of proteins involved in BR biosynthesis and signaling pathways.

**Figure S8. Paclitaxel inhibits axial growth in Arabidopsis.** **A-B.** Representative phenotypes of seedlings at 5 DAG after paclitaxel treatment under light (**A**) and in darkness (**B**). Bar = 0.5 cm. **C.** Root and hypocotyl length under light, n > 12. **D.** Hypocotyl length in darkness, n > 9. Data are means ± SD. Results are representative of three independent experiments. Significant differences were assessed by one-way ANOVA followed by Tukey’s post-hoc test; different letters indicate significant differences (*p* < 0.05).

**Figure S9. Comparison of inhibitory effects of paclitaxel and L2 in Arabidopsis. A.** Representative phenotypes of seedlings at 5 DAG under light after L2 or paclitaxel treatment. Bar = 0.5 cm. **B.** Root length under light in **A**, n > 15. **C.** Representative phenotypes of seedlings at 5 DAG in darkness after L2 or paclitaxel treatment. Bar = 0.5 cm. **D.** Hypocotyl length in darkness in **C**, n > 15. Wild type seedlings were treated with 4 µM L2 or paclitaxel under light or in darkness. **E.** EdU staining of proliferating cells in roots of wild type seedlings at 3 DAG. Bar = 50 μm. **F.** Quantification of EdU incorporation shown as the intensity ratio measured in ImageJ, n > 15. **G.** Western blotting of β-tubulin in total extracts; UGPase and histone H3 were used as cytoplasmic and nuclear loading controls, respectively. 4 independent biological replicates are shown for each treatment. Quantification of β-tubulin protein levels from all 4 biological replicates is shown to the right, normalized to UGPase and relative to mock. Data are mean ± SD; statistical significance was determined by one-way ANOVA followed by Tukey’s post-hoc test, and different letters indicate significant differences (p < 0.05). **H.** Representative cortical microtubule arrays in hypocotyl epidermal cells of the *TUB6-mCherry* line at 5 DAG under light after L2 or paclitaxel treatment. Bar = 10 μm. **I-K.** Quantification of microtubule density (**I**), anisotropy (**J**), and orientation (**K**) from **H**. For (**I**) and (**J**), data are mean ± SD from 3 biological replicates per treatment (≥10 cells per replicate); statistical tests were performed on replicate-level means. Significant differences were assessed by one-way ANOVA followed by Tukey’s post-hoc test; different letters indicate significant differences (p < 0.05). For (**K**) rose diagrams show the frequency distribution of microtubule orientation angles relative to the cell long axis, measured using FibrilTool and binned into 10° intervals (0–180°); >30 cells per treatment from 3 biological replicates are shown for visualization only.

**Figure S10. Comparison of inhibitory effects of L2 and paclitaxel in rice and tobacco. A.** Representative phenotypes of 8-day-old rice seedlings grown under light. Wild type ZH11 seedlings at 4 DAG were treated with 4 μM L2 or paclitaxel for 4 days. Bar = 2 cm. **B**-**C.** Root and shoot lengths of rice seedlings, n > 15. **D.** Representative phenotype of 11-day-old tobacco seedlings grown under light. Seedlings at 2 DAG were treated with 4 μM L2 or paclitaxel for 9 days. **E.** Root length of tobacco seedlings, n > 39. Data are means ± SD. Results are representative of three independent experiments, significant differences were assessed by one-way ANOVA followed by Tukey’s post-hoc test, different letters indicate significant differences (*p* < 0.05).

**Figure S11. L2 inhibits proliferation and motility of rat vascular smooth muscle cells. A.** Representative cell outlines at 24 h. Cells were treated with no drug (Blank), mock (DMSO), and L2 (1, 5, and 10 μM), or paclitaxel (10 μM). **B.** Cell migration trajectories overlaid as blue tracks. **C-D.** Quantification of migration displacement (**C**) and migration speed (**D)**. **E.** Colored polygons showing cell masks at 0 and 24 h. **F.** Quantification of cell proliferation. Data are means ± SE, n = 3 biological replicates, statistical significance was assessed by one-way ANOVA followed by Tukey’s post-hoc test (**C**, **D**, **F**).

**Figure S12. L2 inhibits growth of the organoids derived from multiple human cancers. A.** Schematic diagram of organoids treated with different concentrations of L2, responding individuals are highlighted. **B.** Representative ovarian cancer organoids after 5-day L2 treatment. Bar = 100 μm. **C.** Representative intrahepatic cholangiocarcinoma organoid after 3-day L2 treatment. Bar = 100 μm. **D.** Representative glioma organoid after 3-day L2 treatment. Bar = 100 μm. Red arrowheads indicate newly detached/free cells at organoid edges in **B-D**. **E.** Ki67 immunostaining with DAPI nuclear staining in cryosections of ovarian cancer organoids. Bar = 50 μm.

**Figure S13. Cell type-resolved expression of proliferation-, apoptosis-, and cell cycle-related genes in OVA-64 organoids following L2 treatment.** Heatmap showing Z-score-normalized expression of curated marker genes representing three functional gene sets-proliferation/viability, apoptosis and cell-cycle regulation-across the eight major cell types identified by snRNA-seq. For each cell type, mean expression is shown separately for the mock control and L2-treated groups. Rows are grouped by functional category and columns are grouped by cell type and treatment condition. Color scale indicates row-wise Z-score, with red representing relatively higher expression and blue representing relatively lower expression within each gene**.**

**Figure S14. Toxicity assessment of L2 in zebrafish embryos. A.** Survival of embryos at 72 h post-fertilization (hpf) after exposure to L2 or paclitaxel at 10, 20, and 40 µM. **B.** Survival of embryos at 96 hpf after exposure to L2 or irinotecan (CPT-11) at 10, 20, and 40 µM. **C.** Representative phenotypic abnormalities at 72 hpf. Drug-exposed embryos exhibit curved tails (black arrows) and pericardial edema (red arrows) compared with mock (DMSO). Statistical significance for survival comparisons was assessed by the log-rank (Mantel-Cox) test (**A**-**B**). ns, not significant; **, *p* < 0.01; ****, *p* < 0.0001.
